# Genotypic and phenotypic comparison of drug resistance profiles of clinical multidrug-resistant *Mycobacterium tuberculosis* isolates using whole genome sequencing in Latvia

**DOI:** 10.1186/s12879-023-08629-7

**Published:** 2023-09-28

**Authors:** Anda Vīksna, Darja Sadovska, Iveta Berge, Ineta Bogdanova, Annija Vaivode, Lauma Freimane, Inga Norvaiša, Iveta Ozere, Renāte Ranka

**Affiliations:** 1 Riga East Clinical University Hospital, Centre of Tuberculosis and Lung Diseases, Ropaži Municipality, Stopiņi Parish, Upeslejas, Latvia; 2 Rīga Stradiņš University, Riga, Latvia; 3 Latvian Biomedical Research and Study Centre, Ratsupites Str. 1, Riga, LV-1067 Latvia

**Keywords:** Drug-resistant tuberculosis, Whole genome sequencing, Drug susceptibility testing, Latvia

## Abstract

**Background:**

Multidrug-resistant tuberculosis (MDR–TB) remains a major public health problem in many high tuberculosis (TB) burden countries. Phenotypic drug susceptibility testing (DST) take several weeks or months to result, but line probe assays and Xpert/Rif Ultra assay detect a limited number of resistance conferring gene mutations. Whole genome sequencing (WGS) is an advanced molecular testing method which theoretically can predict the resistance of *M. tuberculosis* (Mtb) isolates to all anti-TB agents through a single analysis.

**Methods:**

Here, we aimed to identify the level of concordance between the phenotypic and WGS-based genotypic drug susceptibility (DS) patterns of MDR–TB isolates. Overall, data for 12 anti-TB medications were analyzed.

**Results:**

In total, 63 MDR–TB Mtb isolates were included in the analysis, representing 27.4% of the total number of MDR–TB cases in Latvia in 2012–2014. Among them, five different sublineages were detected, and 2.2.1 (Beijing group) and 4.3.3 (Latin American-Mediterranean group) were the most abundant. There were 100% agreement between phenotypic and genotypic DS pattern for isoniazid, rifampicin, and linezolid. High concordance rate (> 90%) between phenotypic and genotypic DST results was detected for ofloxacin (93.7%), pyrazinamide (93.7%) and streptomycin (95.4%). Phenotypic and genotypic DS patterns were poorly correlated for ethionamide (agreement 56.4%), ethambutol (85.7%), amikacin (82.5%), capreomycin (81.0%), kanamycin (85.4%), and moxifloxacin (77.8%). For capreomycin, resistance conferring mutations were not identified in several phenotypically resistant isolates, and, in contrary, for ethionamide, ethambutol, amikacin, kanamycin, and moxifloxacin the resistance-related mutations were identified in several phenotypically sensitive isolates.

**Conclusions:**

WGS is a valuable tool for rapid genotypic DST for all anti-TB agents. For isoniazid and rifampicin phenotypic DST potentially can be replaced by genotypic DST based on 100% agreement between the tests. However, discrepant results for other anti-TB agents limit their prescription based solely on WGS data. For clinical decision, at the current level of knowledge, there is a need for combination of genotypic DST with modern, validated phenotypic DST methodologies for those medications which did not showed 100% agreement between the methods.

**Supplementary Information:**

The online version contains supplementary material available at 10.1186/s12879-023-08629-7.

## Background

Tuberculosis (TB) remains a major public health problem and is still one of the main causes of death worldwide; in 2021, there were 1.6 million TB-related deaths [1]. Rifampicin and multidrug resistant tuberculosis (RR/MDR–TB) is a burden for healthcare system, mainly because the duration of treatment is longer than drug susceptible TB. The World Health Organization (WHO) reported that in 2021 there were 10.6 million people ill with TB, and 450,000 of them had RR/MDR–TB, which accounts to 3.6% of all new TB cases and 18% of the previously treated ones [1]. Drug susceptibility testing (DST) of *Mycobacterium tuberculosis* (Mtb) is crucial for clinicians to choose the most appropriate treatment for every individual TB patient, especially for MDR–TB cases. Phenotypic culture-based drug susceptibility tests have been the DST gold standard for a long time, but it takes several weeks or months to obtain the result; moreover, for some drugs, inappropriately high breakpoints have resulted in systematic false-susceptible DST results [2, 3]. Molecular-based DST as line probe assays and the Xpert MTB/Rif assay are available in clinical laboratories and are widely used, however, these tests detect a limited number of gene mutations and do not show heteroresistance [2, 4].

The advanced molecular drug resistance detection method is based on the whole genome sequencing (WGS), which theoretically can predict the resistance of Mtb isolates to all anti-TB agents through a single analysis [5]. Although the use of WGS analysis increases, its value is still limited due to incomplete databases which are used to prescribe predicted mutations for resistance of drugs, and due to lack of knowledge about all resistance-associated mutations [6]. Several databases have been developed recently specifically for analyzing Mtb WGS data, such as TB profiler, KvarQ, TGS-TB, CASTB, PhyResSe, MTBseq, and ReSeqTB-UVP [7]. Recently, based on systematic analysis of a large collection of Mtb isolates with WGS and phenotypic DST, WHO team has developed the high-quality, comprehensive catalogue of confidence-graded Mtb genetic markers [8]. For the isoniazid and rifampicin, WGS-based molecular drug resistance tests reach 91.3–97.5% sensitivity and 93.6–99.0% specificity [8, 9]. On the other hand, according to the literature data, the genotype-phenotype correlation remains low for pyrazinamide, ethambutol, ethionamide, and fluoroquinolones [6, 10, 11]. Thus, simultaneous phenotypic and genotypic drug resistance analysis of Mtb isolates is of a great importance.

In Latvia, a Baltic state in Northern Europe, TB therapy is applied following the relevant WHO guidelines and according to the DST data of patient’s isolates. During the last decade, the total number of reported TB cases in the country had decreased. For example, in 2012 there were 880 TB cases (43 per 100,000), and 101 (11.5%) of them were RR/MDR–TB cases. But current situation is better: in 2021 only 261 TB cases (13.8 per 100,000) were reported, and 25 (9.6%) of them were MDR–TB [12]. Nevertheless, the proportion of MDR–TB cases in Latvia remains high, thus Mtb drug resistance studies are of a high importance. Here, we aimed to identify the level of concordance between the phenotypic DST data of Latvian MDR–TB isolates with the mutation profiles obtained by application of WGS analysis.

## Materials and methods

### Sample collection

For this study, Mtb isolates were obtained from stock cultures of clinical isolates at the Riga East Clinical University Hospital, Centre of Tuberculosis and Lung Diseases of Latvia. Samples collected from 2012 to 2014 with confirmed phenotypic resistance to at least isoniazid and rifampicin were included. Second sample selection criterion was the availability of additional phenotypic DST data for, at least, ethambutol, amikacin, ofloxacin or levofloxacin, and pyrazinamide. One patient was represented with one isolate. The Mtb isolates and corresponding DNA samples were anonymized by code.

Phenotypic DST of anti-TB drugs was carried out based on WHO technical manual for DST of medicines used in the treatment of TB [13, 14]. Phenotypic DST was performed using BACTEC MGIT 960 system (MGIT) and/or Lowenstein-Jensen (LJ) solid media for the following drugs: isoniazid, rifampicin, ethambutol, pyrazinamide, streptomycin, amikacin, kanamycin, capreomycin, ofloxacin, moxifloxacin, ethionamide, and linezolid. For this study, phenotypic data were critically reviewed to ensure matching the current WHO recommendations [14]. Kanamycin and capreomycin are no longer endorsed for TB treatment, however, they were included in the current study for historical interest and for additional interpretation of mutations that confer resistance to amikacin. Similarly, ofloxacin testing is no longer recommended; however, in our study, ofloxacin was tested in WHO-endorsed media at concentrations x (2.0 µg/ml MGIT and 4.0 µg/ml LJ) which are equivalent to testing levofloxacin at concentrations x/2 (1.0 µg/ml MGIT and 2.0 µg/ml LJ). For this reason, according to the WHO, phenotypic DST results for ofloxacin could be used to represent levofloxacin resistance [8, 14].

### **Mycobacterium tuberculosis*DNA samples, WGS and further sequencing data processing***

Mtb isolate DNA was extracted using GenoLyse kit (Hain Lifescience, Germany) according to manufacturer’s protocol. All mycobacterial DNA samples were purified using Nucleomag magnetic beads (Macherey-Nagel, Netherlands) in ratio 1:2 prior to library preparation. DNA was eluted in 10 mM Tris buffer solution (pH = 8; AppliChem, Germany). Paired-end fragment libraries were prepared using the QIAseq FX DNA library kit (Qiagen, Germany), and sequenced on a MiSeq platform (Illumina, US). Reads of maximum 600 bp were produced.

Sequencing data were processed by using appropriate bioinformatics tools and pipelines. Bioinformatic operations were conducted on the Galaxy web platform [15]. The created pipeline was based on the one available at the Galaxy community hub [16]. The Trimmomatic tool (v0.38.0) was used for adapter sequences, low-quality ends (Phred quality score < 20), and sliding window trimming (average Phred quality score of 20 across the 10-base period), keeping sequences of at least 20 base pairs long. Outputs were mapped to the reference sequence using the snippy tool (v4.6.0). The genome of the inferred Mtb complex’s most recent common ancestor combined with the annotation of the H37Rv sequence (GenBank NC000962.3) was used as a reference [17]. Left aligning of indels was performed by BamLeftAlign (v1.3.6), while BAM filter (v0.5.9) was used to keep only properly paired mapped reads, and remove sequences marked as PCR duplicates. Generated BAM file was used as an input for the TB-Profiler tool (v4.1.1) which discriminated lineages and detected resistance associated mutations in studied isolates. Further, any detected changes in resistance-related genes were manually checked against the most recent WHO mutation catalogue data (WHO, 2021). Only mutations with allele frequency ≥ 10% supported by at least four sequencing reads were called. Mutation grading was applied based on WHO mutation catalogue data [8]. Genotyping DST result was assumed to be “Resistant” if group 1 “Associated with resistance”, group 2 “Associated with resistance, interim” or group 3 “Uncertain significance” mutations were detected. Genotyping DST result was assumed to be “Sensitive” if group 4 “Not associated with resistance, interim”, group 5 “Not associated with resistance”, or non-graded mutations were detected.

Additional amplification of Mtb *pncA* gene was performed to confirm indels detected by WGS. Polymerase chain reaction (PCR) was conducted based on previously published protocol [18]. Forward 5′-AACAGTTCATCCCGGTTC-3′ and reverse 5′-GCGTCATGGACCCTATATC-3′ primers targeting the whole gene (product length – 668 bp) were used. The PCR mixture (26 µL) was prepared as follows: 13 µL of 2x DreamTaq PCR Master Mix (Thermo Fisher Scientific, US), 0.2 µM of each primer, adding 2 µL of DNA template. Thermal cycling conditions were 95 °C for 3 min followed by 35 cycles of 30 s at 95 °C, 30 s at 58 °C, 1 min at 72 °C, and a final extension of 72 °C for 5 min. PCR product was visualized on 1.5% agarose gel (TopVision Agarose, Thermo Fisher Scientific, US). Sanger sequencing of positive amplicons was performed using BrilliantDye Terminator (v3.1) Cycle Sequencing Kit (NimaGen, Netherlands) on ABI Prism 3100 Genetic Analyzer (PerkinElmer, USA).

## Results

### Genotyping analysis of drug resistant isolates

In total, 63 Mtb isolates were obtained, representing approximately 1/3 of the total number of MDR–TB cases in Latvia in the study period (63/230 cases, 27.4%, years 2012–2014). Among them, five different sublineages were detected (Table 1). According to the WGS-based data, more than a half of all MDR–TB samples belonged to the sublineage 2.2.1 (Beijing group, 34/63, 54.0%). The second most abundant sublineage was 4.3.3 (Latin American-Mediterranean (LAM) group, 23/63, 36.5%). Other sublineages were detected in much lower proportions: 4.2.1 (Ural) was detected in three cases (4.8%), 4.8 - in two cases (3.2%), and 4.3.3/4.2.1 sublineage was represented by only one isolate (1.6%).

**Table 1:** Phenotypic and genotypic antimicrobial resistance in multidrug-resistant *M. tuberculosis* isolates

*M. tuberculosis* sublineage	Method	Anti-tuberculosis drug											
		INH	STM	RIF	EMB	AMK	KAN	CAP	OFX	MFX	PZA	ETO	LZD
2.2.1 (N = 34)	pDST, n (%)	34/34 (100.0)	22/23 (95.7)	34/34 (100)	30/34 (88.2)	12/34 (35.3)	9/25 (36.0)	12/34 (35.3)	10/34 (29.4)	1/6 (16.7)	26/34 (76.5)	8/26 (30.8)	0/4 (0.0)
	gDST, n (%)	34/34 (100.0)	34 /34 (100.0)	34/34 (100.0)	33/34 (97.1)	23/34 (67.7)	23/34 (67.7)	11/34 (32.4)	9/34 (26.5)	9/34 (26.5)	25/34 (73.5)	25/34 (73.5)	0/34 (0.0)
	Match, n (%)	34/34 (100)	22/23 (95.7)	34/34 (100)	29/34 (85.3)	23/34 (67.7)	19/25 (76.0)	33/34 (97.1)	31/34 (91.2)	4/6 (66.7)	31/34 (91.2)	16/26 (61.5)	4/4 (100.0)
4.3.3 (N = 23)	pDST, n (%)	23/23 (100.0)	17/17 (100.0)	23/23 (100.0)	21/23 (91.3)	12/23 (52.2)	6/13 (46.2)	22/23 (95.7)	6/23 (26.1)	2/9 (22.2)	22/23 (95.7)	5/11 (45.5)	0/4 (0.0)
	gDST, n (%)	23/23 (100.0)	23/23 (100.0)	23/23 (100.0)	23/23 (100.0)	12/23 (52.2)	12/23 (52.2)	12/23 (52.2)	7/23 (30.4)	7/23 (30.4)	21/23 (91.3)	23/23 (100.0)	0/23 (0.0)
	Match, n (%)	23/23 (100)	17/17 (100.0)	23/23 (100.0)	21/23 (91.3)	23/23 (100.0)	13/13 (100.0)	13/23 (56.5)	22/23 (95.7)	7/9 (77.7)	22/23 (95.7)	5/11 (45.5)	4/4 (100.0)
4.2.1 (N = 3)	pDST, n (%)	3/3 (100.0)	1/1 (100.0)	3/3 (100.0)	2/3 (66.7)	0/3 (0.0)	0/1 (0.0)	0/3 (0.0)	0/3 (0.0)	0/2 (0.0)	2/3 (66.7)	0/1 (0.0)	0/2 (0.0)
	gDST, n (%)	3/3 (100.0)	3/3 (100.0)	3/3 (100.0)	2/3 (66.7)	0/3 (0.0)	0/3 (0.0)	0/3 (0.0)	0/3 (0.0)	0/3 (0.0)	2/3 (66.7)	2/3 (66.7)	0/3 (0.0)
	Match, n (%)	3/3 (100)	1/1 (100.0)	3/3 (100)	3/3 (100.0)	3/3 (100.0)	1/1 (100.0)	3/3 (100.0)	3/3 (100.0)	2/2 (100.0)	3/3 (100.0)	0/1 (0.0)	3/3 (0.0)
4.8 (N = 2)	pDST, n (%)	2/2 (100.0)	1/1 (100.0)	2/2 (100.0)	1/2 (50.0)	0/2 (0.0)	0/1 (0.0)	0/2 (0.0)	0/2 (0.0)	0/1 (0.0)	2/2 (100.0)	na	0/1 (0.0)
	gDST, n (%)	2/2 (100.0)	0/2 (0.0)	2/2 (100.0)	2/2 (100.0)	0/2 (0.0)	0/2 (0.0)	0/2 (0.0)	0/2 (0.0)	0/1 (0.0)	2/2 (100.0)	2/2 (100.0)	0/2 (0.0)
	Match, n (%)	2/2 (100.0)	0/1 (0.0)	2/2 (100.0)	1/2 (50.0)	2/2 (100.0)	1/1 (100.0)	2/2 (100.0)	2/2 (100.0)	1/1 (100.0)	2/2 (100.0)	na	1/1 (100.0)
4.3.3/4.2.1 (N = 1)	pDST, n (%)	1/1 (100.0)	0/1 (0.0)	1/1 (100.0)	0/1 (0.0)	0/1 (0.0)	0/1 (0.0)	1/1 (100.0)	0/1 (0.0)	na	1/1 (100.0)	1/1 (100.0)	na
	gDST, n (%)	1/1 (100.0)	0/1 (0.0)	1/1 (100.0)	1/1 (100.0)	0/1 (0.0)	0/1 (0.0)	0/1 (0.0)	0/1 (0.0)	0/1 (0.0)	1/1 (100.0)	1/1 (100.0)	0/1 (0.0)
	Match, n (%)	1/1 (100.0)	1/1 (100.0)	1/1 (100.0)	0/1 (0.0)	1/1 (100.0)	1/1 (100.0)	0/1 (0.0)	1/1 (100.0)	na	1/1 (100.0)	1/1 (100.0)	na
Total (N = 63)	pDST, n (%)	63/63 (100.0)	41/43 (95.4)	63/63 (100.0)	54/63 (85.7)	24/63 (38.1)	15/41 (36.6)	35/63 (55.6)	16/63 (25.4)	3/18 (16.7)	53/63 (84.1)	14/39 (35.9)	0/11 (0.0)
	gDST, n (%)	63/63 (100.0)	60 (95.2)	63/63 (100.0)	61/63 (96.8)	35/63 (55.6)	35/63 (55.6)	23/63 (36.5)	16/63 (25.4)	16/63 (25.4)	51/63 (80.9)	53/63 (84.1)	0/63 (0.0)
	Match, n (%)	63/63 (100.0)	41/43 (95.4)	63/63 (100.0)	54/63 (85.7)	52/63 (82.5)	35/41 (85.4)	51/63 (81.0)	59/63 (93.7)	14/18 (77.8)	59/63 (93.7)	22/39 (56.4)	11/11 (100.0)

Abbreviations: pDST, phenotypic drug susceptibility testing; gDST, genotypic drug susceptibility testing; na, not available; INH, isoniazid; STM, streptomycin; RIF, rifampicin; EMB, ethambutol; AMK, amikacin; KAN, kanamycin; CAP, capreomycin; OFX, ofloxacin; MFX, moxifloxacin; PZA, pyrazinamide; ETO, ethionamide; LZD, linezolide

### Phenotypic drug resistance

Phenotypic resistance data were available for 12 anti-TB medications, however, not all Mtb isolates were tested for all drugs, and two methods (LJ media and MGIT) were used interchangeably (Table 1; Supplementary Table 1). According to the phenotypic DST, in addition to isoniazid and rifampicin, MDR Mtb isolates were resistant to streptomycin (95.4%), ethambutol (85.7%), amikacin (38.1%), kanamycin (36.6%), capreomycin (55.6%), ofloxacin (25.4%), moxifloxacin (16.7%), pyrazinamide (84.1%), and ethionamide (35.9%). Resistance to linezolid was tested in 11 isolates; the results showed that all of them were linezolid-susceptible.

### Detection of resistance-associated genetic variants (RAVs)

**Fig. 1:**
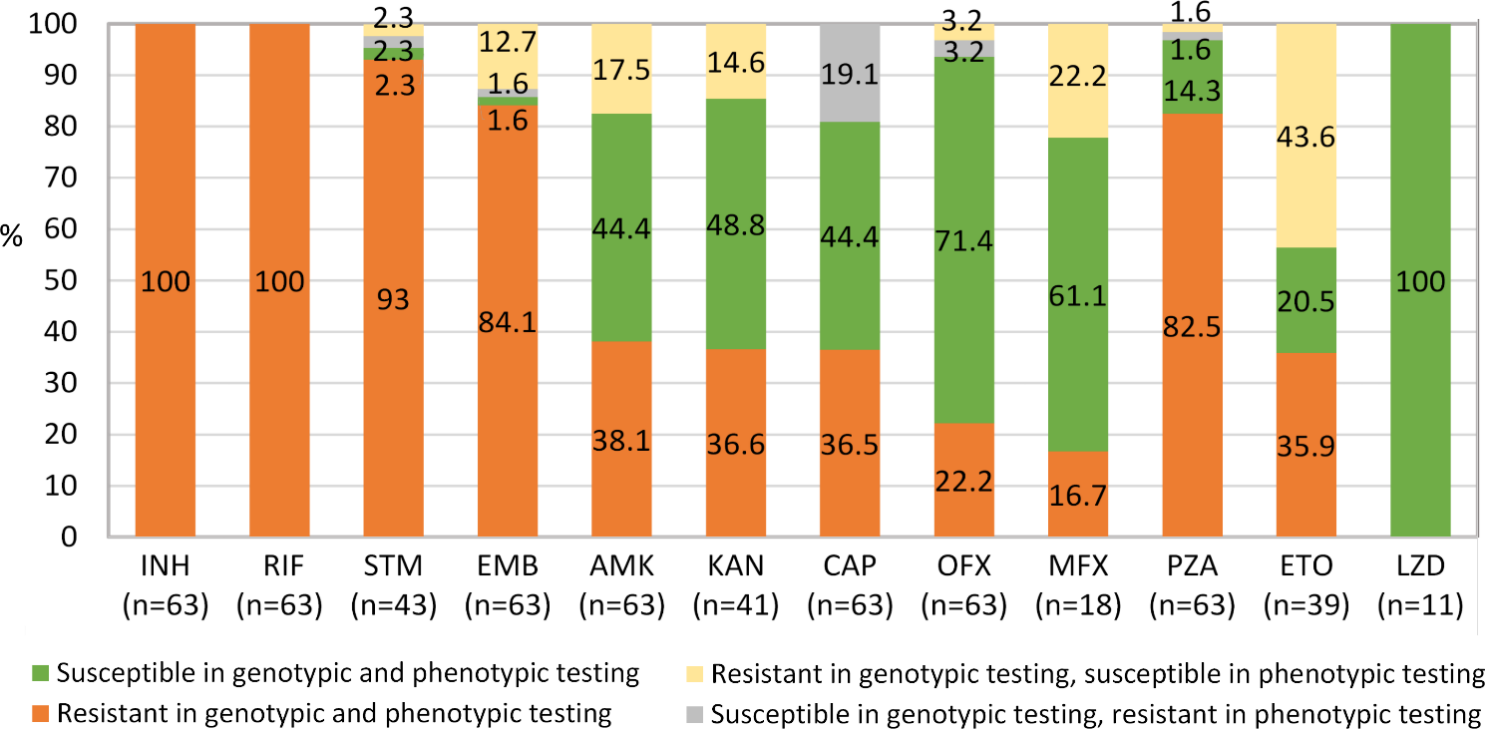
Concordance of the phenotypic and whole genome sequencing-based genotypic drug susceptibility testing results of the multidrug resistant *M. tuberculosis* isolates. Number of tested isolates for each drug is indicated. Abbreviations: INH, isoniazid; STM, streptomycin; RIF, rifampicin; ETO, ethambutol; AMK, amikacin; KAN, kanamycin; CAP, capreomycin; OFX, ofloxacin; PZA, pyrazinamide; ETO, ethionamide; LZD, linezolide

All drug resistance-associated genetic variants (RAVs), which were identified in this study, are summarized in the Supplementary Table 2. Both phenotypic DST and genotypic DST data, as well as the analysis of the relationship between genotypic and phenotypic drug resistance profiles, are provided in Table 1; Fig. 1, as well as in Supplementary Tables 1 and 2.

.

#### Isoniazid, rifampicin, ethionamide

High confidence group 1 RAVs in *katG* and *rpoB* genes, which were linked to resistance to isoniazid and rifampicin, respectively, were detected in all 63 isolates, thus the correlation of phenotypic/genotypic resistance data for both medications was 100%. In *rpoB* gene, nine different RAVs in five genomic positions were detected; in four lineage 2.2.1 isolates two different *rpoB* RAVs were detected simultaneously, and in two lineage 2.2.1 isolates RAV in *rpoB* gene was accompanied by a mutation in *rpoC* gene.

In contrary, all studied Mtb isolates harbored a single *katG* p.Ser315Thr RAV which determined the resistance to isoniazid. On the other hand, *fabG1* c. -15 C > T, *inhA* gene promoter RAV (group 1 mutation), which has been attributed to the resistance to both isoniazid and ethionamide, was detected in 38 (60.3%) of MDR–TB isolates; among them, 13 were phenotypically-resistant to ethionamide, 8 were ethionamide-sensitive, and the phenotypic DST data for 17 MDR–TB isolates were not available. Also, *fabG1* c.-17G > T RAV (group 3 mutation) was detected in two Mtb isolates; one was phenotypically ethionamide-sensitive, the status of the second one was unknown.

RAVs in *ethA* gene, which previously have also been linked to ethionamide resistance, were detected in 25 (39.7%) isolates; among them, 4 were phenotypically-resistant to ethionamide, 10 were ethionamide-sensitive, and the resistance status of 11 MDR–TB isolates was unknown. Seven different RAVs in *ethA* gene were observed, while *ethA* p.Ile338Ser was detected solely in 4.4.3 isolates. Two RAVs, namely *ethA* c.110-111insA and c.1290del, which are currently not included in WHO confidence grading mutation list, were detected along *fabG1* c.-15 C > T group 1 mutation in four and one isolate, respectively. All five isolates belonged to the 2.2.1 sublineage; three were ethionamide-resistant, one – ethionamide-sensitive, and phenotypic DST data were not available for the fifth isolate. Overall, for ethionamide, phenotypic/genotypic data were poorly correlated, as the match was observed for 56.4% of the phenotypically-tested MDR–TB isolates.

#### Ethambutol

RAVs in *embB* gene, which were linked to resistance to ethambutol, were detected in 61 (96.8%) isolates; overall, ten different RAVs in eight genome positions were observed. In four Mtb isolates two different RAVs occurred simultaneously; all four isolates were phenotypically-resistant. Among the phenotypically-sensitive isolates, ethambutol-related RAVs were detected in eight cases; six isolates harbored group 1 mutations in the e*mbB* gene, and two isolates – group 3 mutation. In contrary, none ethambutol-related RAVs were detected in one phenotypically-resistant 2.2.1 Mtb isolate; the resistance was confirmed using both LJ media (2 µg/ml) and MGIT (5.0 µg/ml) methods.

Discrepancies between phenotypic LJ and MGIT ethambutol DST data were observed in 4 isolates; in all cases, high confidence resistance-associated group 1 mutation was detected, namely p.Met306Ile, p.Met306Val and p.Gln497Arg (2 cases). The overall concordance between phenotypic/genotypic data for ethambutol resistance was 85.7%. RAV *embA* c.-43G > C, which is currently graded by WHO as “uncertain significance” (group 3), was present in nine 2.2.1 Mtb isolates; seven were phenotypically ethambutol-resistant, and two – phenotypically sensitive; therefore, a clear conclusion regarding the effect of this mutation could not be drawn. Two additional group 3 mutations, namely, p.Asp1024Asn and p.Asn296His, were detected in four isolates; however, in all cases additional group 1 mutation was present and all four isolates were phenotypically-resistant.

#### Pyrazinamide

RAVs in *pncA* gene, which were linked to resistance to pyrazinamide, were detected in 53 (84.1%) Mtb isolates. Overall, 20 different RAVs were observed; among them, ten were group 1, seven were group 2, one – group 3 mutation, and two RAVs were not graded in the current WHO resistance mutation list. Overall, in three phenotypically-resistant cases, WHO-classified mutations were not detected; however, two of these isolates harbored non-graded insertion/deletion mutations in *pncA* gene (namely, *pncA* c.157-158insCGATG and c.69-74del). In contrary, only in one case, group 1 mutation (*pncA* p.Ser59Pro) was detected in phenotypically-sensitive Mtb isolate. The overall concordance between phenotypic/genotypic data for pyrazinamide resistance was 93.7%.

#### Streptomycin

Overall, streptomycin resistance-related *rrs* and *rpsL* RAVs were detected in 60 (95.2) of Mtb isolates. Among them, *rpsL* p.Lys43Arg was observed exclusively in sublineage 2.2.1 isolates (N = 34), and *rrs* r.514a > c – in Mtb isolates belonged to sublineages 4.3.3 (N = 23) and 4.2.1 (N = 2). Only in one case, no RAVs were detected in phenotypically-resistant Mtb isolate (sublineage 4.8), while in a single case *rpsL* p.Lys43Arg RAV was detected in phenotypically-sensitive 2.2.1 isolate. Thus, the overall concordance between phenotypic/genotypic data for streptomycin resistance in our study reached 95.4%.

#### Amikacin, Kanamycin, Capreomycin

According to the phenotypic DST data, 24/63 (38.1%), 15/41 (36.6%) and 35/63 (55.6%) isolates were resistant to amikacin, kanamycin and capreomycin, respectively (Table 1). The overall match between phenotypic/genotypic data for amikacin, kanamycin and capreomycin was 82.5%, 85.4% and 81.0%, respectively. The r.1401a > g RAV in *rrs* gene, which was previously linked to amikacin, kanamycin and capreomycin resistance, was detected in 23 (36.5%) isolates; the available phenotypic DST results confirmed phenotypic resistance to the aminoglycosides in all cases.

On the other hand, three different RAVs in *eis* gene (namely, c.-10G > A, c.-14 C > T and c.-37G > T), which were linked to amikacin and/or kanamycin resistance, were detected in 12 (19.1%) Mtb isolates; all of the isolates belonged to the sublineage 2.2.1. However, according to the phenotypic DST data, the vast majority of these samples were phenotypically-sensitive to kanamycin (6 of 8) and amikacin (11 of 12). Moreover, in one kanamycin- and amikacin-sensitive isolate two *eis* gene mutations (c.-14 C > T and c.-37G > T) were detected simultaneously. Similarly, the ambiguity between phenotypic/genotypic DST results for capreomycin were observed: in 12 phenotypically capreomycin-resistant isolates no resistance-related RAVs were detected; all but one these isolates were phenotypically sensitive to amikacin or both amikacin and kanamycin.

#### Fluoroquinolones

Based on the phenotypic DST data, ofloxacin resistance was detected in 16/63 (25.4%) isolates, while 3/18 (16.7%) isolates were moxifloxacin-resistant; all fluoroquinolone-resistant isolates belonged to either 2.2.1. or 4.3.3. sublineages. Discordance in the phenotypic DST results between the two fluoroquinolones were observed in five isolates; all were ofloxacin-resistant and moxifloxacin-sensitive.

Overall, in this study, phenotypic DST results did not absolutely correlate with the occurrence of RAVs; the overall match between phenotypic/genotypic data for ofloxacin was 93.7%, and for moxifloxacin − 77.8%. No fluoroquinolone-related RAVs were found in two ofloxacin-resistant isolates. Fluoroquinolone-related RAVs were detected in 16 (25.4%) isolates; all but one occurred in *gyrA* gene, and five different RAVs in four genome positions were observed; all detected *gyrA* RAVs belonged to the group 1 mutations. Among them, *gyrA* p.Ala90Val RAV was detected in one ofloxacin-sensitive and six ofloxacin-resistant isolates; four of these isolates with available moxifloxacin phenotypic DST data were moxifloxacin-sensitive.

#### Linezolid

In our study, no linezolid-resistant Mtb isolates were detected neither by phenotypic, nor genotypic analysis, thus the correlation of phenotypic/genotypic resistance data for this medication was 100%.

### ***Confirmation of insertion/deletion mutations in*pncA*gene***

**Fig. 2:**
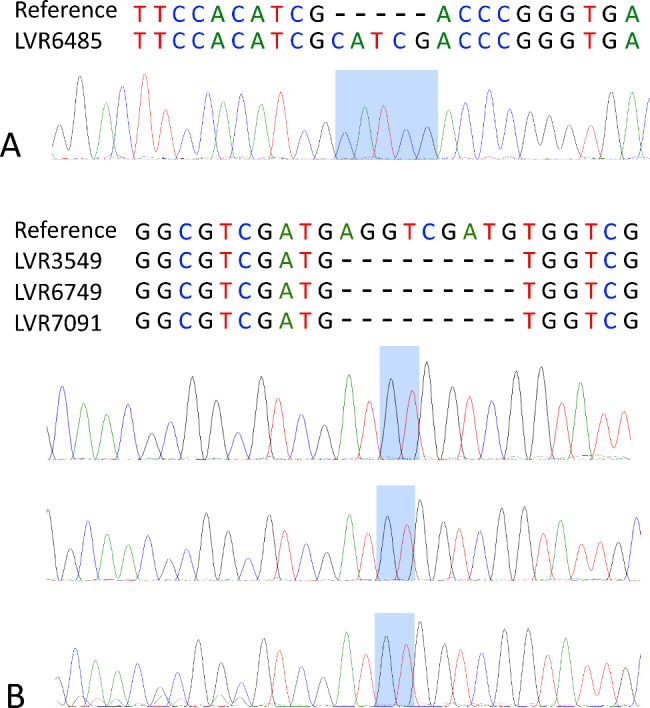
Visual representation of Sanger sequencing analysis of *pncA* gene fragments. The chromatograms presenting insertion/deletion mutations in four *M. tuberculosis* isolates are shown. A: c.157-158insCGATG was detected in one isolate. B: c.380-388del. was detected in three isolates

In our study, WGS analysis revealed insertion/deletion mutations in *pncA* gene. Sanger sequencing analysis was applied to the four isolates in question (harboring mutations *pncA* c.157-158insCGATG and c.380-388del) to rule out the possible sequencing errors. Sanger sequencing results confirmed the presence of mutations in all four isolates (Fig. 2).

## Discussion

This is the first study presenting the results of comparison of the phenotypic and WGS-based genotypic DST results for MDR Mtb isolates in Latvia. Sublineages 2.2.1 and 4.3.3 were the most abundant among drug resistant isolates in our study. This is consistent with the previous study demonstrating that Beijing genotype is common in Latvia, and both Beijing and LAM Mtb isolates were associated with drug resistance more often than other genotypes [19]. Indeed, Beijing lineage strains are globally distributed and are associated with the spread of MDR–TB in Eurasia [20]. The Beijing lineage has highest prevalence in Asia and Europe, especially in Eastern European countries including Czech Republic, Moldova, Russia, and Ukraine [20–24]. The fact that Beijing strains are the main carriers of MDR–TB and extensively drug-resistant TB in Eastern Europe and Central Asia, and can lead to the occurrence of several serious outbreaks in close geographical areas [25], allows to predict MDR–TB spread in Europe due to increased migration of population. Moreover, surge of TB, along with HIV and COVID-19 feared amid war in Ukraine leading to a regional crisis [26].

There were 100% agreement between phenotypic and WGS - based genotypic DST results for isoniazid, rifampicin and linezolid in our study (Fig. 1; Table 1). Similarly, high concordance between both testing approaches has been demonstrated for isoniazid and rifampicin in other studies [27–30]. These observations potentially allow the replacement of phenotypic DST with WGS analysis for both medications. Regarding linezolid, there are known mutations in *rplC* and *rrl* genes which were associated with the resistance [31, 32]. However, we did not identify any linezolid-resistant Mtb isolates, thus, in this study, the clinically-sound conclusions regarding the interchangeability of phenotypic DST and WGS for linezolid could not be drawn.

Phenotypic and genotypic concordance above 90% was obtained for streptomycin (95.4%), ofloxacin (93.7%) and pyrazinamide (93.7%) (Table 1; Fig. 1). For the other six medications, i.e. ethionamide, ethambutol, moxifloxacin, amikacin, kanamycin, and capreomycin, the phenotypic DST and WGS-based drug susceptibility patterns were poorly correlated as the observed concordance between the methods ranged from 56.4 to 85.7%.

The overall concordance of phenotypic and genotypic DST results for ethambutol was 85.7% However, the variability in the agreement among different spoligotypes was observed ranging from 85.3 to 100%; the lowest value was obtained for the sublineage 2.2.1. Inconsistent results of the phenotypic and genotypic DST agreement for ethambutol were also reported in other studies, ranging from 40% up to 94.6% [27, 29, 30]. RAVs in *embB* gene were identified in 61 (96.8%) of the studied isolates, however, eight (13.1%) of them were phenotypically sensitive (Table 1; Fig. 1). The most common mutation among the isolates with discrepant results occurred at the *embB* gene position 306 (four cases, including p.Met306Val, p.Met306Ile and p.Met306Leu), while in two other cases – at the *embB* gene position 497 (p.Gln497Arg). All these mutations have been graded as high confident resistance-related (group 1), thus it could be assumed that all six Mtb isolates were ethambutol-resistant [8, 33, 34]. This fact could be potentially related to the critical concentration (CC) of ethambutol used for the phenotypic DST, because many *embB* mutations confer minimum inhibitory concentrations (MICs) close to the CC, resulting in poor agreement between genotypic and phenotypic DST [30, 35]. In contrary, none currently known ethambutol resistance-related RAVs were detected in a single phenotypically-resistant isolate (Table 1; Fig. 1), and the majority of isolates harbouring solely group 3 mutation *embA* c.-43G > C (7/9, 77.8%) were phenotypically-resistant. The discrepant results betwen phenotypic and genotypic DST for ethambutol deserve further investigation including studies on the correlation with treatment outcome.

Phenotypic DST and WGS data poorly correlated for ethionamide in our study; the concordant results were obtained only for approximately half of Mtb isolates for which both phenotypic and genotypic data were available (56.4%). For this medication the resistance rate detected by WGS was significantly higher compared to the phenotypic testing using LJ media (i.e. 84.1% vs. 35.9%); in contrary, the study of Faksri and colleagues reported moderate concordance rates (81.37%) mainly due to isolates that were phenotypically resistant on Middlebrook 7H10 agar plates but WGS-based susceptible [6]. In our study the discrepant results were observed for 17 phenotypically-sensitive MDR Mtb isolates where genotypic DST ethionamide resistance status was assigned based on the detection of a wide variety of resistance-associated mutations in *fabG1* and/or *ethA* genes. In ten phenotypically-sensitive cases (58.8%), group 1 mutations (*fabG1* c.-15 C > T (n = 8) and *ethA* c.110del (n = 2)) were detected; moreover, in one case *fabG1* c.-15 C > T coincided with the group 3 mutation (*ethA* p.Ile338Ser). In six cases (35.3%) group 2 mutation was detected (*ethA* c.1029del (n = 5), *ethA* c.768del (n = 1)), and the group 3 mutation (*fabG1* c.-17G > T) was detected in one case. These results could be explained by the fact that many ethionamide resistance mutations confer only modest increases in MIC [36]. These results suggest that, currently, mutation test results should be carefully evaluated, because *ethA* and *fabG1* mutant strains could still be sensitive to ethionamide and may be effective for ethionamide treatment. This finding is in line with a study by Song and colleagues [37].

Phenotypic and genotypic DST results concordance for pyrazinamide reached 93.7% in our study, which is similar to the other reported results where the match for 91 − 93.8% of cases was observed [21, 27]. Phenotypic pyrazinamide resistance of our MDR–TB isolates was slightly higher compared to that predicted by WGS (84.1% vs. 80.9%, respectively), because in several isolates no WHO-graded mutations were observed. On the other hand, in one phenotypically-sensitive isolate a high confidence RAV in the *pncA* gene was detected. This observation could be explained by well documented complexity and challenges associated with pyrazinamide in vitro susceptibility testing leading to either false-positive or false-negative results [38]. For pyrazinamide resistance detection WHO recommended genotypic DST assay includes mutation analysis solely in *pncA* gene, however, there are other RAVs reported in genes such as *panD* and *rpsA* [39–41]. Currently, several pyrazinamide resistance-related genes are included in Mtb databases used for WGS data analysis; nevertheless, in our study, only RAVs in the *pncA* gene were detected including 9-nucleotide deletion mutation (c.380-388del) in three isolates. In addition, two different, currently WHO non-graded insertion/deletion mutations (*pncA* c.69-74del and c.157-158insCGATG) were observed in two phenotypically-resistant isolates (one isolate each).

For injectables variable concordance results for phenotypic and genotypic DST were identified, being high for streptomycin (95.4%), and considerably lower for amikacin (82.5%), kanamycin (85.4%), and capreomycin (81.0%). In contrary, in other studies, a full concordance between phenotypic and genotypic DST results for amikacin was reported [11, 30]. Here, all Mtb isolates which had the r.1401a > g RAV in *rrs* gene were characterized as phenotypically cross-resistant either to amikacin and capreomycin, or amikacin, kanamycin and capreomycin; this observation is in agreement with other studies [6, 9, 42]. Overall, the r.1401a > g RAV in *rrs* gene was detected in 23/63 (36.5%) of our isolates. The discordant results for amikacin and kanamycin were mostly because the presence of the mutations in the *eis* gene in phenotypically-sensitive isolates. For example, among 11 isolates harboring solely amikacin group 3 *eis* gene mutations (namely, *eis* c.-10G > A, n = 9; *eis* c.-37G > T, n = 2) only one isolate was phenotypically amikacin-resistant. On the other hand, the same RAVs have been reported as a group 1 mutations for kanamycin; however, in our eight isolates with available phenotypic DST data, only two were kanamycin-resistant. Indeed, up-regulation of *eis* gene due to the promoter mutations has been shown to confer a low-level kanamycin resistance, and, to a lesser extent, increased amikacin MIC [43, 44]. In addition, one amikacin and kanamycin phenotypically-sensitive isolate had both *eis* c.-14 C > T and c.-37G > T mutations. Recently, the study by Vargas and colleagues demonstrated that the *eis* C-14T promoter mutation cannot confer resistance to amikacin and kanamycin if it coincides with loss-of-function mutations in the coding region of *eis* [45]. However, whether the interaction of different *eis* promoter mutations may result in drug susceptibility remains to be deciphered. In contrary, for capreomycin, all discordant isolates were resistant according to the phenotypic testing, but drug-sensitive according to the WGS data.

Our results showed that there was also a high correlation for streptomycin (95.4%) between phenotypic and WGS-based data. However, some studies reported a moderate agreement for streptomycin between both methods [2, 46]. In our study, all streptomycin-resistant sublineage 2.2.1 isolates had a single *rpsL* gene mutation p.Lys43Arg; in contrary, all but one streptomycin-resistant Mtb isolates of other sublineages harbored r.514a > c RAV in *rrs* gene, while p.Lys88Arg RAV in *rpsL* gene was detected in the single sublineage 4.2.1 isolate.

Overall match between phenotypic DST and WGS-based genotypic DST results for ofloxacin was 93.7% in this study; a full concordance was reported in other studies [11, 30]. All ofloxacin/fluoroquinolones-resistant strains belonged to sublineages 2.2.1 and 4.3.3. Two 2.2.1 Mtb isolates were ofloxacin-sensitive according to the phenotypic DST data, while no specific RAVs were detected; however, one of these isolates was moxifloxacin-sensitive based on phenotypic DST results. Another phenotypic/genotypic discordance case could be explained by the presence of a group 3 mutation (*gyrB* Arg446His, uncertain significance), which was detected in the ofloxacin-sensitive sublineage 4.3.3 isolate. In the fourth discordant case, *gyrA* Ala90Val RAV was detected in ofloxacin-sensitive isolate; for this mutation MIC equal to the CC have been reported, thus methodological variation in MIC testing likely accounted for these results [8, 14]. It is usually assumed that all detected RAVs in *gyrA* and *gyrB* genes provide Mtb cross-resistance against several fluoroquinolones, however, in our study, some discordant results were obtained. According to the phenotypic DST data, four Mtb isolates were ofloxacin-resistant and moxifloxacin-sensitive; in all these cases *gyrA* Ala90Val RAV was detected. These results were also the main reason for lower phenotypic/genotypic data match for moxifloxacin. Additional MIC data are required to decipher if the observed mutation was associated with high or low MIC to moxifloxacin.

Our study has several limitations. First, the study period included years 2012–2014. The reason for the chosen study period was the availability of high quality stock cultures of MDR–TB isolates which were thoroughly tested for the phenotypic resistance against 12 anti-TB medications. While not all 63 Mtb isolates were tested for all drugs, phenotypic resistance data for isoniazid, rifampicin, ethambutol, amikacin, capreomycin, ofloxacin, and pyrazinamide were available for all samples. It should be also noted that since 2021 the laboratory of Centre of Tuberculosis and Lung Diseases of Latvia is using Xpert MTB/XDR which allows fast molecular DST for isoniazid, ethionamide, amikacin, kanamycin, capreomycin, and fluoroquinolones. For rifampicin, Xpert/Rif Ultra is used, while bedaquiline, linezolid, delamanid, clofazimine are tested using the BACTEC MGIT solution. However, for studied isolates, bedaquiline, clofazimine and delamanid resistance data were not available and thus were not included in study, and the linezolid-resistant isolates were lacking. However, this study represents a comprehensive analysis of both phenotypic and WGS-based MDR–TB resistance data which are highly relevant not only for our country, but also for other countries with a high burden of MDR–TB.

In conclusion, the results obtained in this study highlight WGS as a valuable tool for prediction of drug resistance in Mtb isolates. However, the observed discordance between phenotypic DST and WGS data for several medications requires further investigation to identify all resistance conferring gene mutations and correlate them with modern, MIC-based phenotypic DST results and treatment outcome. The endemic presence of Mtb Beijing and LAM genotypes associated with drug resistance, as well as the variability of circulating resistance-associated genetic variants, indicates the need to maintain MDR–TB strain data collection, and to continue MDR–TB studies in Latvia. Currently, for clinical decision, the discrepant results for several anti-TB agents limit their prescription based solely on WGS data, while challenges associated with phenotypic DST, especially on LJ solid media, could lead to either false-positive or false-negative results. Thus, for these medications, a combination of genotypic DST with modern, validated phenotypic DST methodologies is required for accurate detection of drug resistance.

### Electronic supplementary material

Below is the link to the electronic supplementary material.

Supplementary Material 1

Supplementary Material 2

## Data Availability

The raw sequencing reads obtained in the study were deposited in European Nucleotide Archive (ENA) under the project accession number PRJEB59824.

## Abbreviations

CC: critical concentration
MIC: minimal inhibitory concentration
TB: tuberculosis
MDR–TB: multidrug resistant tuberculosis
Mtb: *M. tuberculosis*
DST: drug susceptibility testing
WHO: World Health Organization
WGS: whole genome sequencing
