# Supplementary material for: Genotypic and phenotypic comparison of drug resistance profiles of clinical multidrug-resistant Mycobacterium tuberculosis isolates using whole genome sequencing in Latvia
Source: BMC Infect Dis. 2023 Sep 28;23:638. doi: 10.1186/s12879-023-08629-7 (PMC10540372; doi:10.1186/s12879-023-08629-7)
Supplement: Supplementary file 1 — Supplementary Material 1 [file 12879_2023_8629_MOESM1_ESM.pdf]

Supplementary Table 1. *M. tuberculosis* phenotypic drug susceptibility testing (DST) results.

Result description: 1: resistant; 0: sensitive; na: not available.
Cases when discordance with WGS-based genotypic drug susceptibility results were observed are indicated by letter (d)
Cases when the evaluation of agreement between phenotypic/genotypic drug susceptibility testing results was based on non-classified insertion/deletion mutations are indicated by letters (NC); genetic DST result was assumed to be "Sensitive".
Cases when the evaluation of agreement between phenotypic/genotypic drug susceptibility testing results was based on grade 3 mutations (i.e. "uncertain significance") are indicated by letters (g3); genetic DST result was assumed to be "Resistant".
Abbreviations: na, not available; AMK, amikacin; CAP, capreomycin; EMB, ethambutol; ETO, ethionamide; KAN, kanamycin; LZD, linezolid; MFX, moxifloxacin; RIF, rifampicin; STM, streptomycin; PZA, pyrazinamide

ID	Sublineage	INH MGIT 0.1 µg/ml	INH LJ 0.2 µg/ml	RIF MGIT 1.0 µg/ml	RIF LJ 40.0 µg/ml	EMB MGIT 5.0 µg/ml	EMB LJ 2.0 µg/ml	AMK MGIT 1.0 µg/ml	AMK LJ 30.0 µg/ml	KAN LJ 30.0 µg/ml	CAP MGIT 2.5 µg/ml	CAP LJ 40.0 µg/ml	STM MGIT 1.0 µg/ml	STM LJ 4.0 µg/ml	OFX MGIT 2.0 µg/ml	OFX LJ 4.0 µg/ml	MFX MGIT 0.25 µg/ml	PZA MGIT 100.0 µg/ml	ETO LJ 40.0 µg/ml	LZD MGIT 1.0 µg/ml
LVR5354	4.8	1	na	1	na	1	na	0	na	na	0	na	1 (d)	na	0	na	na	1	na	na
LVR0416	4.8	na	1	na	1	na	0 (d)	0	na	0	0	na	na	na	0	0	0	1	na	0
LVR1241	2.2.1	1	na	1	na	1	na	1	na	na	1	na	1	na	1 (d)	na	0	1	na	na
LVR7282	2.2.1	na	1	na	1	na	1	1	1	1	1	1	na	1	0	0	na	0	0	na
LVR6485	2.2.1	na	1	na	1	na	1	na	1	1	na	1	na	1	0	0	na	1 (d, NC)	1	na
LVR9459	2.2.1	1	1	1	1	1 (g3)	1 (g3)	0 (d, g3)	0 (d, g3)	0 (d)	0	0	1	1	1	1	na	1	1	na
LVR2256	2.2.1	1	1	1	1	1	1	0	0	0	0	0	1	1	0	0	na	0	0 (d)	na
LVR4857	2.2.1	1	1	1	1	1	1	0	0	0	0	0	1	1	0	0	na	0	0 (d)	na
LVR3102	2.2.1	1	1	1	na	1	1	0 (d)	0 (d)	0 (d)	0	0	1	1	1	1	na	0 (d)	0 (d)	na
LVR2894	2.2.1	1	1	1	1	1	1	1	1	1	1	1	1	1	0	0	na	1	0 (d)	na
LVR1545	2.2.1	1	1	1	1	0 (d)	0 (d)	0	0	0	0	0	1	1	0	0	na	0	0 (d)	na
LVR4327	2.2.1	na	1	na	1	na	1	0	0	0	0	0	na	1	0	0	na	1 (d)	0	na
LVR1517	2.2.1	1	na	1	na	1	na	0 (d, g3)	na	na	0	na	1	na	1	na	na	1	na	na
LVR1167	2.2.1	na	1	na	1	na	1	na	0 (d, g3)	1	na	0	na	1	1	1	na	1	0 (d)	na
LVR4347	2.2.1	na	1	na	1	na	1	na	1	1	na	1	na	1	0	0	na	1	1	na
LVR0347	2.2.1	1	na	1	na	1	na	1	na	na	1	na	1	na	1 (d)	na	na	1	na	na
LVR9613	2.2.1	1	1	1	1	1	0	na	0	0	na	0	na	na	0	0	na	0	0 (d)	na
LVR3430	2.2.1	na	1	na	1	na	0 (d)	0	0	0	0	0	na	na	na	0	na	0	0	na
LVR8534	2.2.1	1	na	1	na	1	na	1	na	na	1	na	na	na	0	na	0	1	na	0
LVR6570	2.2.1	1	1	1	1	0	1	0	0	0	0	0	na	na	0	0	na	0	0	0
LVR5311	2.2.1	1	1	1	1	1 (g3)	1 (g3)	0 (d, g3)	0 (d, g3)	0 (d)	0	0	na	na	0	0	0	1	1	0
LVR2498	2.2.1	1	1	1	1	0 (d, g3)	0 (d, g3)	0 (d, g3)	0 (d, g3)	0 (d)	0	0	na	na	1	1	0 (d)	1	1	na
LVR8758	2.2.1	1	1	1	1	1	1	1	1	1	1	1	na	na	0	0	na	1	0 (d)	na
LVR2506	2.2.1	na	1	na	1	na	1 (g3)	0 (d, g3)	0 (d, g3)	0 (d)	0	0	na	1	1	1	0 (d)	1	0 (d)	na
LVR5434	2.2.1	na	1	na	1	na	1	0	0	0	0	0	na	na	na	0	na	1	0	na
LVR1552	2.2.1	1	1	1	1	1 (g3)	1 (g3)	0 (d, g3)	0 (d, g3)	1	0	0	na	na	0	0	na	1	1	na
LVR0142	2.2.1	1	na	1	na	1	na	0 (d, g3)	na	na	0	na	1	na	0	na	na	1	na	na
LVR5462	2.2.1	1	na	1	na	1	na	0	na	na	0	0	1	na	0	0	na	1	0	na
LVR3549	2.2.1	1	1	1	1	1 (d)	1 (d)	0	0	0	0	0	1	1	0	0	na	1	0	na
LVR6749	2.2.1	na	1	na	1	na	0 (d, g3)	0	0	0	0	0	na	0 (d)	0	0	na	1	0	na
LVR7091	2.2.1	na	1	na	1	na	1	na	1	1	na	1	na	1	1	1	na	1	0 (d)	na
LVR8181	2.2.1	1	na	1	na	1 (g3)	na	1 (g3)	na	na	1 (d)	na	1	na	0 (d)	na	na	1	na	na
LVR6217	2.2.1	1	na	1	na	1	na	1	na	na	1	na	1	na	0	na	na	1	na	na
LVR7111	2.2.1	1	1	1	1	1 (g3)	1 (g3)	0 (d, g3)	0 (d, g3)	0 (d)	0	0	1	1	0	0	na	1	1	na
LVR9980	2.2.1	1	1	na	1	1	1	1	1	1	1	1	na	na	1	1	1	1	1	0
LVR8846	2.2.1	1	na	1	na	1 (g3)	na	0 (d, g3)	na	na	0	na	na	na	0	na	na	1	na	na
LVR3904	4.2.1	1	na	1	na	0	na	0	na	na	0	na	1	na	0	na	na	0	na	na
LVR0611	4.2.1	1	na	1	na	1	na	0	na	na	0	na	na	na	0	na	0	1	na	0
LVR0008	4.2.1	1	1	1	1	1	1	0	0	0	0	0	na	na	0	0	0	1	0 (d, g3)	0
LVR0781	4.3.3	1	na	1	na	1	na	1	na	na	1	na	1	na	0	na	na	1	na	na
LVR3482	4.3.3	1	na	1	na	1	na	1	na	na	1	na	1	na	0	na	na	1	na	na
LVR9247	4.3.3	na	1	na	1	na	1	0	0	0	1 (d)	1 (d)	na	1	0	0	na	1	0 (d)	na
LVR8303	4.3.3	1	na	1	na	1	na	1	na	na	1	na	1	na	0	na	na	1	na	na
LVR9466	4.3.3	1	1	1	1	1	1	1	1	1	1	1	1	1	0	0	na	1 (g3)	1	na
LVR9469	4.3.3	1	1	1	1	1	0	0	0	0	1 (d)	1 (d)	1	1	0	0	na	1	1	na
LVR0206	4.3.3	1	1	1	1	0	1	0	0	0	1 (d)	1	1	1	0	0	na	1	1	na
LVR4228	4.3.3	1	na	1	na	0 (d)	na	0	na	na	1	na	1	na	0	na	na	1	na	na
LVR0589	4.3.3	1	na	1	na	1	na	1	na	na	1	na	1	na	0 (d, g3)	na	na	1	na	na
LVR9696	4.3.3	1	1	1	1	1	1	1	1	1	1	0	1	1	1	1	na	1	1	na
LVR5695	4.3.3	1	na	1	na	1	na	0	na	na	0	na	na	na	0	na	0	0	na	0
LVR2713	4.3.3	1	na	1	na	1	na	0	na	na	1 (d)	na	na	na	0	na	0	1	na	0
LVR6495	4.3.3	1	1	1	1	1	1	0	0	0	1 (d)	1 (d)	na	na	1	1	1	1	0 (d)	na
LVR3891	4.3.3	1	na	1	na	1	na	1	na	1	1	na	1	na	1	na	1	1	na	na
LVR2827	4.3.3	1	1	1	1	1	1	1	1	1	1	1	1	1	1	1	0 (d)	1	0 (d)	na
LVR3274	4.3.3	1	1	1	1	1	1	0	0	0	1 (d)	1 (d)	1	1	0	0	na	1	0 (d)	na
LVR1189	4.3.3	na	1	na	1	na	1	1	1	1	1	1	na	1	0	0	na	1 (d, NC)	0 (d)	na
LVR0758	4.3.3	na	1	na	1	na	1	na	0	0	na	1 (d)	na	1	na	0	na	1	0 (d)	na
LVR1491	4.3.3	1	na	1	na	0 (d)	na	1	na	1	1	na	1	na	1	na	0	1	na	na
LVR1816	4.3.3	1	na	1	na	1	na	1	na	na	1	na	1	na	0	na	0	1 (g3)	na	na
LVR2221	4.3.3	1	na	1	na	1	na	1	na	na	1	na	na	na	0	na	0	1	na	0
LVR3470	4.3.3	1	1	1	1	1	1	0	0	0	1 (d)	1 (d)	na	na	0	0	0	1	1	0
LVR9235	4.3.3	1	na	1	na	1	na	0	na	na	1 (d)	na	na	na	1	na	na	1	na	na
LVR8377	4.3.3/4.2.1	na	1	na	1	na	0 (d)	na	0	0	na	1 (d)	na	0	0	0	na	1	1	na
Phenotypic/genotypic match, n/tested (%)*		INH		RIF		EMB		AMK		KAN	CAP		STM		OFX		MFX	PZA	ETO	LZD
		63/63 (100.0)		63/63 (100.0)		54/63 (85.7)		52/63 (82.5)		35/41 (85.4)	51/63 (81.0)		41/43 (95.4)		59/63 (93.7)		14/18 (77.8)	59/63 (93.7)	22/39 (56.4)	11/11 (100.0)

* For calculation purposes the results for both phenotypic drug susceptibility testing methods (LJ and MGIT) were combined.
