# Supplementary material for: Genotypic and phenotypic comparison of drug resistance profiles of clinical multidrug-resistant Mycobacterium tuberculosis isolates using whole genome sequencing in Latvia
Source: BMC Infect Dis. 2023 Sep 28;23:638. doi: 10.1186/s12879-023-08629-7 (PMC10540372; doi:10.1186/s12879-023-08629-7)
Supplement: Supplementary file 2 — Supplementary Material 2 [file 12879_2023_8629_MOESM2_ESM.pdf]

Supplementary Table 2. List of drug resistance mutations detected in *M. tuberculosis* isolates.

Mutation grading was assigned based on the WHO mutation catalogue.
(Catalogue of mutations in *Mycobacterium tuberculosis* complex and their association with drug resistance.Geneva: World Health Organization; 2021.)
For aminoglycosides and ethionamide/isoniazid mutations, pDST data are drug-specific.
Abbreviations: pDST, phenotypic drug susceptibility testing; gDST, genotypic drug susceptibility testing.
NA, not available; NC, not classified; AMK, amikacin; KAN, kanamycin; CAP, capreomycin; INH, isoniazid; ETO, ethionamide.

ID	Sublineage	Drug	Genome position	LocusTag	Gene	Mutation	Mutation grading	pDST resistant	Match between pDST/gDST data
LVR0781	4.3.3	rifampicin	761155	Rv0667	rpoB	p.Ser450Leu	1) Assoc w R	yes	yes
LVR0781	4.3.3	streptomycin	1472359	rrs	rrs	r.514a>c	1) Assoc w R	yes	yes
LVR0781	4.3.3	aminoglycosides	1473246	rrs	rrs	r.1401a>g	1) Assoc w R (AMK, KAN, CAP)	yes (AMK, CAP), NA (KAN)	yes (AMK, CAP), NA (KAN)
LVR0781	4.3.3	ethionamide,isoniazid	1673425	Rv1483	fabG1	c.-15C>T	1) Asoc w R (INH, ETO)	yes (INH), NA (ETO)	yes (INH), NA (ETO)
LVR0781	4.3.3	isoniazid	2155168	Rv1908c	katG	p.Ser315Thr	1) Assoc w R	yes	yes
LVR0781	4.3.3	pyrazinamide	2288868	Rv2043c	pncA	p.Val125Gly	2) Assoc w R - Interim	yes	yes
LVR0781	4.3.3	ethambutol	4247469	Rv3795	embB	p.Tyr319Ser	1) Assoc w R	yes	yes
LVR0781	4.3.3	ethionamide	4326461	Rv3854c	ethA	p.Ile338Ser	3) Uncertain significance	NA	NA
LVR3482	4.3.3	rifampicin	761155	Rv0667	rpoB	p.Ser450Leu	1) Assoc w R	yes	yes
LVR3482	4.3.3	streptomycin	1472359	rrs	rrs	r.514a>c	1) Assoc w R	yes	yes
LVR3482	4.3.3	aminoglycosides	1473246	rrs	rrs	r.1401a>g	1) Assoc w R (AMK, KAN, CAP)	yes (AMK, CAP), NA (KAN)	yes (AMK, CAP), NA (KAN)
LVR3482	4.3.3	ethionamide,isoniazid	1673425	Rv1483	fabG1	c.-15C>T	1) Asoc w R (INH, ETO)	yes (INH), NA (ETO)	yes (INH), NA (ETO)
LVR3482	4.3.3	isoniazid	2155168	Rv1908c	katG	p.Ser315Thr	1) Assoc w R	yes	yes
LVR3482	4.3.3	ethambutol	4247469	Rv3795	embB	p.Tyr319Ser	1) Assoc w R	yes	yes
LVR3482	4.3.3	ethionamide	4326461	Rv3854c	ethA	p.Ile338Ser	3) Uncertain significance	NA	NA
LVR3482	4.3.3	pyrazinamide	2289058	Rv2043c	pncA	p.Pro62Thr	2) Assoc w R - Interim	yes	yes
LVR1241	2.2.1	rifampicin	761109	Rv0667	rpoB	p.Asp435Tyr	1) Assoc w R	yes	yes
LVR1241	2.2.1	rifampicin	761166	Rv0667	rpoB	p.Pro454Ser	3) Uncertain significance	yes	yes
LVR1241	2.2.1	streptomycin	781687	Rv0682	rpsL	p.Lys43Arg	1) Assoc w R	yes	yes
LVR1241	2.2.1	aminoglycosides	1473246	rrs	rrs	r.1401a>g	1) Assoc w R (AMK, KAN, CAP)	yes (AMK, CAP), NA (KAN)	yes (AMK, CAP), NA (KAN)
LVR1241	2.2.1	isoniazid	2155168	Rv1908c	katG	p.Ser315Thr	1) Assoc w R	yes	yes
LVR1241	2.2.1	pyrazinamide	2289016	Rv2043c	pncA	p.Thr76Pro	1) Assoc w R	yes	yes
LVR1241	2.2.1	ethambutol	4247730	Rv3795	embB	p.Gly406Ala	1) Assoc w R	yes	yes
LVR1241	2.2.1	ethionamide	4327441	Rv3854c	ethA	c.32del	2) Assoc w R - Interim	NA	NA
LVR1241	2.2.1	fluoroquinolones						yes (OFX), no (MFX)	no (OFX), yes (MFX)
LVR9247	4.3.3	rifampicin	761155	Rv0667	rpoB	p.Ser450Leu	1) Assoc w R	yes	yes
LVR9247	4.3.3	streptomycin	1472359	rrs	rrs	r.514a>c	1) Assoc w R	yes	yes
LVR9247	4.3.3	ethionamide,isoniazid	1673425	Rv1483	fabG1	c.-15C>T	1) Asoc w R (INH, ETO)	yes (INH), no (ETO)	yes (INH), no (ETO)
LVR9247	4.3.3	isoniazid	2155168	Rv1908c	katG	p.Ser315Thr	1) Assoc w R	yes	yes
LVR9247	4.3.3	pyrazinamide	2289054	Rv2043c	pncA	p.Asp63Gly	2) Assoc w R - Interim	yes	yes
LVR9247	4.3.3	ethambutol	4248003	Rv3795	embB	p.Gln497Arg	1) Assoc w R	yes	yes
LVR9247	4.3.3	capreomycin						yes	no
LVR7282	2.2.1.	rifampicin	761155	Rv0667	rpoB	p.Ser450Phe	1) Assoc w R	yes	yes
LVR7282	2.2.1.	streptomycin	781687	Rv0682	rpsL	p.Lys43Arg	1) Assoc w R	yes	yes
LVR7282	2.2.1.	aminoglycosides	1473246	rrs	rrs	r.1401a>g	1) Assoc w R (AMK, KAN, CAP)	yes (AMK, KAN, CAP)	yes (AMK, KAN, CAP)
LVR7282	2.2.1.	isoniazid	2155168	Rv1908c	katG	p.Ser315Thr	1) Assoc w R	yes	yes
LVR7282	2.2.1.	ethambutol	4247429	Rv3795	embB	p.Met306Val	1) Assoc w R	yes	yes
LVR6485	2.2.1.	rifampicin	761110	Rv0667	rpoB	p.Asp435Val	1) Assoc w R	yes	yes
LVR6485	2.2.1.	streptomycin	781687	Rv0682	rpsL	p.Lys43Arg	1) Assoc w R	yes	yes
LVR6485	2.2.1.	aminoglycosides	1473246	rrs	rrs	r.1401a>g	1) Assoc w R (AMK, KAN, CAP)	yes (AMK, KAN, CAP)	yes (AMK, KAN, CAP)
LVR6485	2.2.1	ethionamide,isoniazid	1673425	Rv1483	fabG1	c.-15C>T	1) Asoc w R (INH, ETO)	yes (INH), yes (ETO)	yes (INH), yes (ETO)
LVR6485	2.2.1.	isoniazid	2155168	Rv1908c	katG	p.Ser315Thr	1) Assoc w R	yes	yes
LVR6485	2.2.1	pyrazinamide	2289084	Rv2043c	pncA	c.157-158insCGATG	NC	yes	no
LVR6485	2.2.1.	ethambutol	4248003	Rv3795	embB	p.Gln497Arg	1) Assoc w R	yes	yes
LVR9459	2.2.1	fluoroquinolones	7570	Rv0006	gyrA	p.Ala90Val	1) Assoc w R (LEV/OFX, MXF)	yes (OFX), NA (MFX)	yes (OFX), NA (MFX)
LVR9459	2.2.1.	rifampicin	761110	Rv0667	rpoB	p.Asp435Val	1) Assoc w R	yes	yes
LVR9459	2.2.1.	streptomycin	781687	Rv0682	rpsL	p.Lys43Arg	1) Assoc w R	yes	yes
LVR9459	2.2.1	ethionamide,isoniazid	1673425	Rv1483	fabG1	c.-15C>T	1) Asoc w R (INH, ETO)	yes (INH), yes (ETO)	yes (INH), yes (ETO)
LVR9459	2.2.1.	isoniazid	2155168	Rv1908c	katG	p.Ser315Thr	1) Assoc w R	yes	yes
LVR9459	2.2.1	pyrazinamide	2288935	Rv2043c	pncA	p.Tyr103His	1) Assoc w R	yes	yes
LVR9459	2.2.1.	kanamycin, amikacin	2715342	Rv2416c	eis	c.-10G>A	1) Assoc w R (KAN); 3) Uncertain significance (AMK)	no (KAN, AMK)	no (KAN, AMK)
LVR9459	2.2.1	ethionamide	4327363	Rv3854c	ethA	c.110-111insA	NC	yes	
LVR9459	2.2.1	ethambutol	4243190	Rv3794	embA	c.-43G>C	3) Uncertain significance	yes	yes
LVR8303	4.3.3.	rifampicin	761155	Rv0667	rpoB	p.Ser450Leu	1) Assoc w R	yes	yes
LVR8303	4.3.3.	streptomycin	1472359	rrs	rrs	r.514a>c	1) Assoc w R	yes	yes
LVR8303	4.3.3.	aminoglycosides	1473246	rrs	rrs	r.1401a>g	1) Assoc w R (AMK, KAN, CAP)	yes (AMK, CAP), NA (KAN)	yes (AMK, CAP), NA (KAN)
LVR8303	4.3.3	ethionamide,isoniazid	1673425	Rv1483	fabG1	c.-15C>T	1) Asoc w R (INH, ETO)	yes (INH), NA (ETO)	yes (INH), NA (ETO)
LVR8303	4.3.3.	isoniazid	2155168	Rv1908c	katG	p.Ser315Thr	1) Assoc w R	yes	yes
LVR8303	4.3.3.	ethambutol	4247469	Rv3795	embB	p.Tyr319Ser	1) Assoc w R	yes	yes
LVR8303	4.3.3	ethionamide	4326461	Rv3854c	ethA	p.Ile338Ser	3) Uncertain significance	NA	NA
LVR8303	4.3.3	pyrazinamide	2289058	Rv2043c	pncA	p.Pro62Thr	2) Assoc w R - Interim	yes	yes
LVR9466	4.3.3.	rifampicin	761110	Rv0667	rpoB	p.Asp435Val	1) Assoc w R	yes	yes
LVR9466	4.3.3.	streptomycin	1472359	rrs	rrs	r.514a>c	1) Assoc w R	yes	yes
LVR9466	4.3.3.	aminoglycosides	1473246	rrs	rrs	r.1401a>g	1) Assoc w R (AMK, KAN, CAP)	yes (AMK, KAN, CAP)	yes (AMK, KAN, CAP)
LVR9466	4.3.3	ethionamide,isoniazid	1673425	Rv1483	fabG1	c.-15C>T	1) Asoc w R (INH, ETO)	yes (INH), yes (ETO)	yes (INH), yes (ETO)
LVR9466	4.3.3.	isoniazid	2155168	Rv1908c	katG	p.Ser315Thr	1) Assoc w R	yes	yes
LVR9466	4.3.3.	ethambutol	4247431	Rv3795	embB	p.Met306Ile	1) Assoc w R	yes	yes
LVR9466	4.3.3	pyrazinamide	2289069	Rv2043c	pncA	p.Phe58Cys	3) Uncertain significance	yes	yes
LVR9469	4.3.3.	rifampicin	761155	Rv0667	rpoB	p.Ser450Leu	1) Assoc w R	yes	
LVR9469	4.3.3.	streptomycin	1472359	rrs	rrs	r.514a>c	1) Assoc w R	yes	yes
LVR9469	4.3.3	ethionamide,isoniazid	1673425	Rv1483	fabG1	c.-15C>T	1) Asoc w R (INH, ETO)	yes (INH), yes (ETO)	yes (INH), yes (ETO)
LVR9469	4.3.3.	isoniazid	2155168	Rv1908c	katG	p.Ser315Thr	1) Assoc w R	yes	yes
LVR9469	4.3.3	pyrazinamide	2289054	Rv2043c	pncA	p.Asp63Gly	2) Assoc w R - Interim	yes	yes
LVR9469	4.3.3.	ethambutol	4248003	Rv3795	embB	p.Gln497Arg	1) Assoc w R	yes	yes
LVR9469	4.3.3.	capreomycin						yes	no
LVR0206	4.3.3.	rifampicin	761155	Rv0667	rpoB	p.Ser450Leu	1) Assoc w R	yes	yes
LVR0206	4.3.3.	streptomycin	1472359	rrs	rrs	r.514a>c	1) Assoc w R	yes	yes
LVR0206	4.3.3	ethionamide,isoniazid	1673425	Rv1483	fabG1	c.-15C>T	1) Asoc w R (INH, ETO)	yes (INH), yes (ETO)	yes (INH), yes (ETO)
LVR0206	4.3.3.	isoniazid	2155168	Rv1908c	katG	p.Ser315Thr	1) Assoc w R	yes	yes
LVR0206	4.3.3	pyrazinamide	2289054	Rv2043c	pncA	p.Asp63Gly	2) Assoc w R - Interim	yes	yes
LVR0206	4.3.3.	ethambutol	4248003	Rv3795	embB	p.Gln497Arg	1) Assoc w R	yes	yes
LVR0206	4.3.3.	capreomycin						yes	no
LVR2256	2.2.1.	rifampicin	761155	Rv0667	rpoB	p.Ser450Leu	1) Assoc w R	yes	yes
LVR2256	2.2.1.	streptomycin	781687	Rv0682	rpsL	p.Lys43Arg	1) Assoc w R	yes	yes
LVR2256	2.2.1.	isoniazid	2155168	Rv1908c	katG	p.Ser315Thr	1) Assoc w R	yes	yes
LVR2256	2.2.1.	ethambutol	4247429	Rv3795	embB	p.Met306Val	1) Assoc w R	yes	yes
LVR2256	2.2.1	ethionamide	4326444	Rv3854c	ethA	c.1029del	2) Assoc w R - Interim	no	no
LVR3904	4.2.1	rifampicin	761110	Rv0667	rpoB	p.Asp435Val	1) Assoc w R	yes	yes
LVR3904	4.2.1	streptomycin	781822	Rv0682	rpsL	p.Lys88Arg	1) Assoc w R	yes	yes

LVR3904	4.2.1	isoniazid	2155168	Rv1908c	katG	p.Ser315Thr	1) Assoc w R	yes	yes
LVR5354	4.8	rifampicin	761139	Rv0667	rpoB	p.His445Asp	1) Assoc w R	yes	yes
LVR5354	4.8	ethionamide,isoniazid	1673425	Rv1483	fabG1	c.-15C>T	1) Asoc w R (INH, ETO)	yes (INH), NA (ETO)	yes (INH), NA (ETO)
LVR5354	4.8	isoniazid	2155168	Rv1908c	katG	p.Ser315Thr	1) Assoc w R	yes	yes
LVR5354	4.8	pyrazinamide	2289057	Rv2043c	pncA	p.Pro62Leu	1) Assoc w R	yes	yes
LVR5354	4.8	ethambutol	4247431	Rv3795	embB	p.Met306Ile	1) Assoc w R	yes	yes
LVR5354	4.8	streptomycin						yes	no
LVR4857	2.2.1.	rifampicin	761155	Rv0667	rpoB	p.Ser450Leu	1) Assoc w R	yes	yes
LVR4857	2.2.1.	streptomycin	781687	Rv0682	rpsL	p.Lys43Arg	1) Assoc w R	yes	yes
LVR4857	2.2.1.	isoniazid	2155168	Rv1908c	katG	p.Ser315Thr	1) Assoc w R	yes	yes
LVR4857	2.2.1.	ethambutol	4247429	Rv3795	embB	p.Met306Val	1) Assoc w R	yes	yes
LVR4857	2.2.1	ethionamide	4326444	Rv3854c	ethA	c.1029del	2) Assoc w R - Interim	no	no
LVR3102	2.2.1	fluoroquinolones	7570	Rv0006	gyrA	p.Ala90Val	1) Assoc w R (LEV/OFX, MXF)	yes (OFX), NA (MFX)	yes (OFX), NA (MFX)
LVR3102	2.2.1	fluoroquinolones	7572	Rv0006	gyrA	p.Ser91Pro	1) Assoc w R (LEV/OFX, MXF)	yes (OFX), NA (MFX)	yes (OFX), NA (MFX)
LVR3102	2.2.1.	rifampicin	761155	Rv0667	rpoB	p.Ser450Leu	1) Assoc w R	yes	yes
LVR3102	2.2.1.	rifampicin	761161	Rv0667	rpoB	p.Leu452Pro	1) Assoc w R	yes	yes
LVR3102	2.2.1.	streptomycin	781687	Rv0682	rpsL	p.Lys43Arg	1) Assoc w R	yes	yes
LVR3102	2.2.1.	isoniazid	2155168	Rv1908c	katG	p.Ser315Thr	1) Assoc w R	yes	yes
LVR3102	2.2.1	pyrazinamide	2289067	Rv2043c	pncA	p.Ser59Pro	1) Assoc w R	no	no
LVR3102	2.2.1.	kanamycin, amikacin	2715346	Rv2416c	eis	c.-14C>T	1) Assoc w R (KAN, AMK)	no (KAN, AMK)	no (KAN, AMK)
LVR3102	2.2.1.	kanamycin, amikacin	2715369	Rv2416c	eis	c.-37G>T	1) Assoc w R (KAN); 3) Uncertain significance (AMK)	no (KAN, AMK)	no (KAN, AMK)
LVR3102	2.2.1.	ethambutol	4247429	Rv3795	embB	p.Met306Val	1) Assoc w R	yes	yes
LVR3102	2.2.1	ethionamide	4326705	Rv3854c	ethA	c.768del	2) Assoc w R - Interim	no	no
LVR2894	2.2.1.	rifampicin	761155	Rv0667	rpoB	p.Ser450Leu	1) Assoc w R	yes	yes
LVR2894	2.2.1.	streptomycin	781687	Rv0682	rpsL	p.Lys43Arg	1) Assoc w R	yes	yes
LVR2894	2.2.1.	aminoglycosides	1473246	rrs	rrs	r.1401a>g	1) Assoc w R (AMK, KAN, CAP)	yes (AMK, KAN, CAP)	yes (AMK, KAN, CAP)
LVR2894	2.2.1.	isoniazid	2155168	Rv1908c	katG	p.Ser315Thr	1) Assoc w R	yes	yes
LVR2894	2.2.1	pyrazinamide	2289040	Rv2043c	pncA	p.Trp68Gly	1) Assoc w R	yes	yes
LVR2894	2.2.1.	ethambutol	4247429	Rv3795	embB	p.Met306Val	1) Assoc w R	yes	yes
LVR2894	2.2.1	ethionamide	4326444	Rv3854c	ethA	c.1029del	2) Assoc w R - Interim	no	no
LVR4228	4.3.3	rifampicin	761155	Rv0667	rpoB	p.Ser450Leu	1) Assoc w R	yes	yes
LVR4228	4.3.3	streptomycin	1472359	rrs	rrs	r.514a>c	1) Assoc w R	yes	yes
LVR4228	4.3.3	ethionamide,isoniazid	1673425	Rv1483	fabG1	c.-15C>T	1) Asoc w R (INH, ETO)	yes (INH), NA (ETO)	yes (INH), NA (ETO)
LVR4228	4.3.3	isoniazid	2155168	Rv1908c	katG	p.Ser315Thr	1) Assoc w R	yes	yes
LVR4228	4.3.3	pyrazinamide	2289054	Rv2043c	pncA	p.Asp63Gly	2) Assoc w R - Interim	yes	yes
LVR4228	4.3.3	ethambutol	4248003	Rv3795	embB	p.Gln497Arg	1) Assoc w R	no	no
LVR4228	4.3.3	capreomycin						yes	no
LVR1545	2.2.1	rifampicin	761155	Rv0667	rpoB	p.Ser450Leu	1) Assoc w R	yes	yes
LVR1545	2.2.1	streptomycin	781687	Rv0682	rpsL	p.Lys43Arg	1) Assoc w R	yes	yes
LVR1545	2.2.1	isoniazid	2155168	Rv1908c	katG	p.Ser315Thr	1) Assoc w R	yes	yes
LVR1545	2.2.1	ethambutol	4247429	Rv3795	embB	p.Met306Val	1) Assoc w R	no	no
LVR1545	2.2.1	ethionamide	4326444	Rv3854c	ethA	c.1029del	2) Assoc w R - Interim	no	no
LVR0589	4.3.3	fluoroquinolones	6576	Rv0005	gyrB	p.Arg446His	3) Uncertain significance (LEV, MXF)	yes (OFX), NA (MFX)	yes (OFX), NA (MFX)
LVR0589	4.3.3	rifampicin	761155	Rv0667	rpoB	p.Ser450Leu	1) Assoc w R	yes	yes
LVR0589	4.3.3	streptomycin	1472359	rrs	rrs	r.514a>c	1) Assoc w R	yes	yes
LVR0589	4.3.3	aminoglycosides	1473246	rrs	rrs	r.1401a>g	1) Assoc w R (AMK, KAN, CAP)	yes (AMK, CAP), NA (KAN)	yes (AMK, CAP), NA (KAN)
LVR0589	4.3.3	ethionamide,isoniazid	1673425	Rv1483	fabG1	c.-15C>T	1) Asoc w R (INH, ETO)	yes (INH), NA (ETO)	yes (INH), NA (ETO)
LVR0589	4.3.3	isoniazid	2155168	Rv1908c	katG	p.Ser315Thr	1) Assoc w R	yes	yes
LVR0589	4.3.3	ethambutol	4247469	Rv3795	embB	p.Tyr319Ser	1) Assoc w R	yes	yes
LVR0589	4.3.3	ethionamide	4326461	Rv3854c	ethA	p.Ile338Ser	3) Uncertain significance	NA	NA
LVR0589	4.3.3	pyrazinamide	2289058	Rv2043c	pncA	p.Pro62Thr	2) Assoc w R - Interim	yes	yes
LVR4327	2.2.1	rifampicin	761155	Rv0667	rpoB	p.Ser450Leu	1) Assoc w R	yes	yes
LVR4327	2.2.1	streptomycin	781687	Rv0682	rpsL	p.Lys43Arg	1) Assoc w R	yes	yes
LVR4327	2.2.1	isoniazid	2155168	Rv1908c	katG	p.Ser315Thr	1) Assoc w R	yes	yes
LVR4327	2.2.1	ethambutol	4247429	Rv3795	embB	p.Met306Val	1) Assoc w R	yes	yes
LVR4327	2.2.1	pyrazinamide						yes	no
LVR1517	2.2.1	fluoroquinolones	7582	Rv0006	gyrA	p.Asp94Gly	1) Assoc w R (LEV/OFX, MXF)	yes (OFX), NA (MFX)	yes (OFX), NA (MFX)
LVR1517	2.2.1	rifampicin	761110	Rv0667	rpoB	p.Asp435Val	1) Assoc w R	yes	yes
LVR1517	2.2.1	streptomycin	781687	Rv0682	rpsL	p.Lys43Arg	1) Assoc w R	yes	yes
LVR1517	2.2.1	ethionamide,isoniazid	1673425	Rv1483	fabG1	c.-15C>T	1) Asoc w R (INH, ETO)	yes (INH), NA (ETO)	yes (INH), NA (ETO)
LVR1517	2.2.1	isoniazid	2155168	Rv1908c	katG	p.Ser315Thr	1) Assoc w R	yes	yes
LVR1517	2.2.1	pyrazinamide	2288988	Rv2043c	pncA	p.Leu85Arg	1) Assoc w R	yes	yes
LVR1517	2.2.1	kanamycin, amikacin	2715369	Rv2416c	eis	c.-37G>T	1) Assoc w R (KAN); 3) Uncertain significance (AMK)	NA (KAN), no (AMK)	NA (KAN), no (AMK)
LVR1517	2.2.1	ethambutol	4247429	Rv3795	embB	p.Met306Val	1) Assoc w R	yes	yes
LVR1167	2.2.1	fluoroquinolones	7582	Rv0006	gyrA	p.Asp94Gly	1) Assoc w R (LEV/OFX, MXF)	yes (OFX), NA (MFX)	yes (OFX), NA (MFX)
LVR1167	2.2.1	rifampicin	761110	Rv0667	rpoB	p.Asp435Val	1) Assoc w R	yes	yes
LVR1167	2.2.1	streptomycin	781687	Rv0682	rpsL	p.Lys43Arg	1) Assoc w R	yes	yes
LVR1167	2.2.1	ethionamide,isoniazid	1673425	Rv1483	fabG1	c.-15C>T	1) Asoc w R (INH, ETO)	yes (INH), no (ETO)	yes (INH), no (ETO)
LVR1167	2.2.1	isoniazid	2155168	Rv1908c	katG	p.Ser315Thr	1) Assoc w R	yes	yes
LVR1167	2.2.1	pyrazinamide	2288988	Rv2043c	pncA	p.Leu85Arg	1) Assoc w R	yes	yes
LVR1167	2.2.1	kanamycin, amikacin	2715369	Rv2416c	eis	c.-37G>T	1) Assoc w R (KAN); 3) Uncertain significance (AMK)	yes (KAN), no (AMK)	yes (KAN), no (AMK)
LVR1167	2.2.1	ethambutol	4247429	Rv3795	embB	p.Met306Val	1) Assoc w R	yes	yes
LVR4347	2.2.1	rifampicin	761110	Rv0667	rpoB	p.Asp435Val	1) Assoc w R	yes	yes
LVR4347	2.2.1	streptomycin	781687	Rv0682	rpsL	p.Lys43Arg	1) Assoc w R	yes	yes
LVR4347	2.2.1	aminoglycosides	1473246	rrs	rrs	r.1401a>g	1) Assoc w R (AMK, KAN, CAP)	yes (AMK, KAN, CAP)	yes (AMK, KAN, CAP)
LVR4347	2.2.1	ethionamide,isoniazid	1673425	Rv1483	fabG1	c.-15C>T	1) Asoc w R (INH, ETO)	yes (INH), yes (ETO)	yes (INH), yes (ETO)
LVR4347	2.2.1	isoniazid	2155168	Rv1908c	katG	p.Ser315Thr	1) Assoc w R	yes	yes
LVR4347	2.2.1	pyrazinamide	2288920	Rv2043c	pncA	p.Gly108Arg	2) Assoc w R - Interim	yes	yes
LVR4347	2.2.1	ethambutol	4247431	Rv3795	embB	p.Met306Ile	1) Assoc w R	yes	yes
LVR4347	2.2.1	ethionamide	4326183	Rv3854c	ethA	c.1290del	NC	yes	
LVR9696	4.3.3	fluoroquinolones	7570	Rv0006	gyrA	p.Ala90Val	1) Assoc w R (LEV/OFX, MXF)	yes (OFX), NA (MFX)	yes (OFX), NA (MFX)
LVR9696	4.3.3	rifampicin	761155	Rv0667	rpoB	p.Ser450Leu	1) Assoc w R	yes	yes
LVR9696	4.3.3	streptomycin	1472359	rrs	rrs	r.514a>c	1) Assoc w R	yes	yes
LVR9696	4.3.3	aminoglycosides	1473246	rrs	rrs	r.1401a>g	1) Assoc w R (AMK, KAN, CAP)	yes (AMK, KAN, CAP)	yes (AMK, KAN, CAP)
LVR9696	4.3.3	ethionamide,isoniazid	1673425	Rv1483	fabG1	c.-15C>T	1) Asoc w R (INH, ETO)	yes (INH), yes (ETO)	yes (INH), yes (ETO)
LVR9696	4.3.3	isoniazid	2155168	Rv1908c	katG	p.Ser315Thr	1) Assoc w R	yes	yes
LVR9696	4.3.3	pyrazinamide	2289206	Rv2043c	pncA	p.Asp12Glu	1) Assoc w R	yes	yes
LVR9696	4.3.3	ethambutol	4247429	Rv3795	embB	p.Met306Leu	1) Assoc w R	yes	yes
LVR8377	4.3.3/4.2.1	rifampicin	761155	Rv0667	rpoB	p.Ser450Leu	1) Assoc w R	yes	yes
LVR8377	4.3.3/4.2.1	ethionamide,isoniazid	1673425	Rv1483	fabG1	c.-15C>T	1) Asoc w R (INH, ETO)	yes (INH), yes (ETO)	yes (INH), yes (ETO)
LVR8377	4.3.3/4.2.1	isoniazid	2155168	Rv1908c	katG	p.Ser315Thr	1) Assoc w R	yes	yes
LVR8377	4.3.3/4.2.1	pyrazinamide	2289054	Rv2043c	pncA	p.Asp63Gly	2) Assoc w R - Interim	yes	yes
LVR8377	4.3.3/4.2.1	ethambutol	4248003	Rv3795	embB	p.Gln497Arg	1) Assoc w R	no	no
LVR8377	4.3.3/4.2.1	capreomycin						yes	no
LVR0347	2.2.1	rifampicin	761155	Rv0667	rpoB	p.Ser450Leu	1) Assoc w R	yes	yes
LVR0347	2.2.1	streptomycin	781687	Rv0682	rpsL	p.Lys43Arg	1) Assoc w R	yes	yes
LVR0347	2.2.1	aminoglycosides	1473246	rrs	rrs	r.1401a>g	1) Assoc w R (AMK, KAN, CAP)	yes (AMK, CAP), NA (KAN)	yes (AMK, CAP), NA (KAN)
LVR0347	2.2.1	isoniazid	2155168	Rv1908c	katG	p.Ser315Thr	1) Assoc w R	yes	yes
LVR0347	2.2.1	pyrazinamide	2289040	Rv2043c	pncA	p.Trp68Gly	1) Assoc w R	yes	yes
LVR0347	2.2.1	ethambutol	4247429	Rv3795	embB	p.Met306Val	1) Assoc w R	yes	yes

LVR0347	2.2.1	ethionamide	4326444	Rv3854c	ethA	c.1029del	2) Assoc w R - Interim	NA	NA
LVR0347	2.2.1	fluoroquinolones						yes (OFX), NA (MFX)	no (OFX), NA (MFX)
LVR9613	2.2.1	rifampicin	761155	Rv0667	rpoB	p.Ser450Leu	1) Assoc w R	yes	yes
LVR9613	2.2.1	rifampicin	764841	Rv0668	rpoC	p.Ile491Thr	NC	yes	yes
LVR9613	2.2.1	streptomycin	781687	Rv0682	rpsL	p.Lys43Arg	1) Assoc w R	NA	NA
LVR9613	2.2.1	isoniazid	2155168	Rv1908c	katG	p.Ser315Thr	1) Assoc w R	yes	yes
LVR9613	2.2.1	ethambutol	4247431	Rv3795	embB	p.Met306Ile	1) Assoc w R	yes	yes
LVR9613	2.2.1	ethionamide	4327363	Rv3854c	ethA	c.110del	1) Assoc w R	no	no
LVR5695	4.3.3	rifampicin	761155	Rv0667	rpoB	p.Ser450Leu	1) Assoc w R	yes	yes
LVR5695	4.3.3	streptomycin	1472359	rrs	rrs	r.514a>c	1) Assoc w R	NA	NA
LVR5695	4.3.3	ethionamide,isoniazid	1673425	Rv1483	fabG1	c.-15C>T	1) Asoc w R (INH, ETO)	yes (INH), NA (ETO)	yes (INH), NA (ETO)
LVR5695	4.3.3	isoniazid	2155168	Rv1908c	katG	p.Ser315Thr	1) Assoc w R	yes	yes
LVR5695	4.3.3	ethambutol	4247469	Rv3795	embB	p.Tyr319Ser	1) Assoc w R	yes	yes
LVR0416	4.8	rifampicin	761139	Rv0667	rpoB	p.His445Asp	1) Assoc w R	yes	yes
LVR0416	4.8	ethionamide,isoniazid	1673425	Rv1483	fabG1	c.-15C>T	1) Asoc w R (INH, ETO)	yes (INH), NA (ETO)	yes (INH), NA (ETO)
LVR0416	4.8	isoniazid	2155168	Rv1908c	katG	p.Ser315Thr	1) Assoc w R	yes	yes
LVR0416	4.8	pyrazinamide	2289057	Rv2043c	pncA	p.Pro62Leu	1) Assoc w R	yes	yes
LVR0416	4.8	ethambutol	4247431	Rv3795	embB	p.Met306Ile	1) Assoc w R	no	no
LVR3430	2.2.1	rifampicin	761155	Rv0667	rpoB	p.Ser450Leu	1) Assoc w R	yes	yes
LVR3430	2.2.1	streptomycin	781687	Rv0682	rpsL	p.Lys43Arg	1) Assoc w R	NA	NA
LVR3430	2.2.1	isoniazid	2155168	Rv1908c	katG	p.Ser315Thr	1) Assoc w R	yes	yes
LVR3430	2.2.1	ethambutol	4247431	Rv3795	embB	p.Met306Ile	1) Assoc w R	no	no
LVR8534	2.2.1	rifampicin	761155	Rv0667	rpoB	p.Ser450Phe	1) Assoc w R	yes	yes
LVR8534	2.2.1	streptomycin	781687	Rv0682	rpsL	p.Lys43Arg	1) Assoc w R	NA	NA
LVR8534	2.2.1	aminoglycosides	1473246	rrs	rrs	r.1401a>g	1) Assoc w R (AMK, KAN, CAP)	yes (AMK, CAP), NA (KAN)	yes (AMK, CAP), NA (KAN)
LVR8534	2.2.1	isoniazid	2155168	Rv1908c	katG	p.Ser315Thr	1) Assoc w R	yes	yes
LVR8534	2.2.1	pyrazinamide	2288826	Rv2043c	pncA	p.Val139Gly	1) Assoc w R	yes	yes
LVR8534	2.2.1	ethambutol	4247429	Rv3795	embB	p.Met306Val	1) Assoc w R	yes	yes
LVR6570	2.2.1	rifampicin	761140	Rv0667	rpoB	p.His445Arg	1) Assoc w R	yes	yes
LVR6570	2.2.1	streptomycin	781687	Rv0682	rpsL	p.Lys43Arg	1) Assoc w R	NA	NA
LVR6570	2.2.1	isoniazid	2155168	Rv1908c	katG	p.Ser315Thr	1) Assoc w R	yes	yes
LVR6570	2.2.1	ethambutol	4247429	Rv3795	embB	p.Met306Val	1) Assoc w R	yes	yes
LVR5311	2.2.1	rifampicin	761110	Rv0667	rpoB	p.Asp435Val	1) Assoc w R	yes	yes
LVR5311	2.2.1	streptomycin	781687	Rv0682	rpsL	p.Lys43Arg	1) Assoc w R	NA	NA
LVR5311	2.2.1	ethionamide,isoniazid	1673425	Rv1483	fabG1	c.-15C>T	1) Asoc w R (INH, ETO)	yes (INH), yes (ETO)	yes (INH), yes (ETO)
LVR5311	2.2.1	isoniazid	2155168	Rv1908c	katG	p.Ser315Thr	1) Assoc w R	yes	yes
LVR5311	2.2.1	pyrazinamide	2288935	Rv2043c	pncA	p.Tyr103His	1) Assoc w R	yes	yes
LVR5311	2.2.1	kanamycin, amikacin	2715342	Rv2416c	eis	c.-10G>A	1) Assoc w R (KAN); 3) Uncertain significance (AMK)	no (KAN, AMK)	no (KAN, AMK)
LVR5311	2.2.1	ethambutol	4243190	Rv3794	embA	c.-43G>C	3) Uncertain significance	yes	yes
LVR2713	4.3.3	rifampicin	761155	Rv0667	rpoB	p.Ser450Leu	1) Assoc w R	yes	yes
LVR2713	4.3.3	streptomycin	1472359	rrs	rrs	r.514a>c	1) Assoc w R	NA	NA
LVR2713	4.3.3	ethionamide,isoniazid	1673425	Rv1483	fabG1	c.-15C>T	1) Asoc w R (INH, ETO)	yes (INH), NA (ETO)	yes (INH), NA (ETO)
LVR2713	4.3.3	isoniazid	2155168	Rv1908c	katG	p.Ser315Thr	1) Assoc w R	yes	yes
LVR2713	4.3.3	pyrazinamide	2289054	Rv2043c	pncA	p.Asp63Gly	2) Assoc w R - Interim	yes	yes
LVR2713	4.3.3	ethambutol	4248003	Rv3795	embB	p.Gln497Arg	1) Assoc w R	yes	yes
LVR2713	4.3.3	capreomycin						yes	no
LVR0611	4.2.1	rifampicin	761140	Rv0667	rpoB	p.His445Leu	1) Assoc w R	yes	yes
LVR0611	4.2.1	streptomycin	1472359	rrs	rrs	r.514a>c	1) Assoc w R	NA	NA
LVR0611	4.2.1	ethionamide,isoniazid	1673423	Rv1483	fabG1	c.-17G>T	3) Uncertain significance (INH, ETO)	yes (INH), NA (ETO)	yes (INH), NA (ETO)
LVR0611	4.2.1	isoniazid	2155168	Rv1908c	katG	p.Ser315Thr	1) Assoc w R	yes	yes
LVR0611	4.2.1	pyrazinamide	2289111	Rv2043c	pncA	p.Val44Gly	2) Assoc w R - Interim	yes	yes
LVR0611	4.2.1	ethambutol	4247730	Rv3795	embB	p.Gly406Ala	1) Assoc w R	yes	yes
LVR0611	4.2.1	ethambutol	4249583	Rv3795	embB	p.Asp1024Asn	3) Uncertain significance	yes	yes
LVR0008	4.2.1	rifampicin	761140	Rv0667	rpoB	p.His445Leu	1) Assoc w R	yes	yes
LVR0008	4.2.1	streptomycin	1472359	rrs	rrs	r.514a>c	1) Assoc w R	NA	NA
LVR0008	4.2.1	ethionamide,isoniazid	1673423	Rv1483	fabG1	c.-17G>T	3) Uncertain significance (INH, ETO)	yes (INH), no (ETO)	yes (INH), no (ETO)
LVR0008	4.2.1	isoniazid	2155168	Rv1908c	katG	p.Ser315Thr	1) Assoc w R	yes	yes
LVR0008	4.2.1	pyrazinamide	2289111	Rv2043c	pncA	p.Val44Gly	2) Assoc w R - Interim	yes	yes
LVR0008	4.2.1	ethambutol	4247730	Rv3795	embB	p.Gly406Ala	1) Assoc w R	yes	yes
LVR0008	4.2.1	ethambutol	4249583	Rv3795	embB	p.Asp1024Asn	3) Uncertain significance	yes	yes
LVR6495	4.3.3	fluoroquinolones	7581	Rv0006	gyrA	p.Asp94Asn	1) Assoc w R (LEV/OFX, MXF)	yes (OFX), yes (MFX)	yes (OFX), yes (MFX)
LVR6495	4.3.3	rifampicin	761155	Rv0667	rpoB	p.Ser450Leu	1) Assoc w R	yes	yes
LVR6495	4.3.3	streptomycin	1472359	rrs	rrs	r.514a>c	1) Assoc w R	NA	NA
LVR6495	4.3.3	ethionamide,isoniazid	1673425	Rv1483	fabG1	c.-15C>T	1) Asoc w R (INH, ETO)	yes (INH), no (ETO)	yes (INH), no (ETO)
LVR6495	4.3.3	isoniazid	2155168	Rv1908c	katG	p.Ser315Thr	1) Assoc w R	yes	yes
LVR6495	4.3.3	pyrazinamide	2289054	Rv2043c	pncA	p.Asp63Gly	2) Assoc w R - Interim	yes	yes
LVR6495	4.3.3	ethambutol	4248003	Rv3795	embB	p.Gln497Arg	1) Assoc w R	yes	yes
LVR6495	4.3.3	capreomycin						yes	no
LVR2498	2.2.1	fluoroquinolones	7570	Rv0006	gyrA	p.Ala90Val	1) Assoc w R (LEV/OFX, MXF)	yes (OFX), no (MFX)	yes (OFX), no (MFX)
LVR2498	2.2.1	rifampicin	761110	Rv0667	rpoB	p.Asp435Val	1) Assoc w R	yes	yes
LVR2498	2.2.1	streptomycin	781687	Rv0682	rpsL	p.Lys43Arg	1) Assoc w R	NA	NA
LVR2498	2.2.1	ethionamide,isoniazid	1673425	Rv1483	fabG1	c.-15C>T	1) Asoc w R (INH, ETO)	yes (INH), yes (ETO)	yes (INH), yes (ETO)
LVR2498	2.2.1	isoniazid	2155168	Rv1908c	katG	p.Ser315Thr	1) Assoc w R	yes	yes
LVR2498	2.2.1	pyrazinamide	2288935	Rv2043c	pncA	p.Tyr103His	1) Assoc w R	yes	yes
LVR2498	2.2.1	kanamycin, amikacin	2715342	Rv2416c	eis	c.-10G>A	1) Assoc w R (KAN); 3) Uncertain significance (AMK)	no (KAN, AMK)	no (KAN, AMK)
LVR2498	2.2.1	ethionamide	4327363	Rv3854c	ethA	c.110-111insA	NC	yes	
LVR2498	2.2.1	ethambutol	4243190	Rv3794	embA	c.-43G>C	3) Uncertain significance	no	no
LVR3891	4.3.3	fluoroquinolones	7581	Rv0006	gyrA	p.Asp94Tyr	1) Assoc w R (LEV/OFX, MXF)	yes (OFX), yes (MFX)	yes (OFX), yes (MFX)
LVR3891	4.3.3	rifampicin	761155	Rv0667	rpoB	p.Ser450Leu	1) Assoc w R	yes	yes
LVR3891	4.3.3	streptomycin	1472359	rrs	rrs	r.514a>c	1) Assoc w R	yes	yes
LVR3891	4.3.3	aminoglycosides	1473246	rrs	rrs	r.1401a>g	1) Assoc w R (AMK, KAN, CAP)	yes (AMK, KAN, CAP)	yes (AMK, KAN, CAP)
LVR3891	4.3.3	ethionamide,isoniazid	1673425	Rv1483	fabG1	c.-15C>T	1) Asoc w R (INH, ETO)	yes (INH), NA (ETO)	yes (INH), NA (ETO)
LVR3891	4.3.3	isoniazid	2155168	Rv1908c	katG	p.Ser315Thr	1) Assoc w R	yes	yes
LVR3891	4.3.3	pyrazinamide	2289027	Rv2043c	pncA	p.Cys72Tyr	2) Assoc w R - Interim	yes	yes
LVR3891	4.3.3	ethambutol	4247469	Rv3795	embB	p.Tyr319Ser	1) Assoc w R	yes	yes
LVR3891	4.3.3	ethionamide	4326461	Rv3854c	ethA	p.Ile338Ser	3) Uncertain significance	NA	NA
LVR8758	2.2.1	rifampicin	761155	Rv0667	rpoB	p.Ser450Leu	1) Assoc w R	yes	yes
LVR8758	2.2.1	streptomycin	781687	Rv0682	rpsL	p.Lys43Arg	1) Assoc w R	NA	NA
LVR8758	2.2.1	aminoglycosides	1473246	rrs	rrs	r.1401a>g	1) Assoc w R (AMK, KAN, CAP)	yes (AMK, KAN, CAP)	yes (AMK, KAN, CAP)
LVR8758	2.2.1	isoniazid	2155168	Rv1908c	katG	p.Ser315Thr	1) Assoc w R	yes	yes
LVR8758	2.2.1	pyrazinamide	2289040	Rv2043c	pncA	p.Trp68Gly	1) Assoc w R	yes	yes
LVR8758	2.2.1	ethambutol	4247429	Rv3795	embB	p.Met306Val	1) Assoc w R	yes	yes
LVR8758	2.2.1	ethionamide	4326444	Rv3854c	ethA	c.1029del	2) Assoc w R - Interim	no	no
LVR2506	2.2.1	fluoroquinolones	7570	Rv0006	gyrA	p.Ala90Val	1) Assoc w R (LEV/OFX, MXF)	yes (OFX), no (MFX)	yes (OFX), no (MFX)
LVR2506	2.2.1	rifampicin	761110	Rv0667	rpoB	p.Asp435Val	1) Assoc w R	yes	yes
LVR2506	2.2.1	streptomycin	781687	Rv0682	rpsL	p.Lys43Arg	1) Assoc w R	yes	yes
LVR2506	2.2.1	ethionamide,isoniazid	1673425	Rv1483	fabG1	c.-15C>T	1) Asoc w R (INH, ETO)	yes (INH), no (ETO)	yes (INH), no (ETO)
LVR2506	2.2.1	isoniazid	2155168	Rv1908c	katG	p.Ser315Thr	1) Assoc w R	yes	yes
LVR2506	2.2.1	pyrazinamide	2288935	Rv2043c	pncA	p.Tyr103His	1) Assoc w R	yes	yes
LVR2506	2.2.1	kanamycin, amikacin	2715342	Rv2416c	eis	c.-10G>A	1) Assoc w R (KAN); 3) Uncertain significance (AMK)	no (KAN, AMK)	no (KAN, AMK)

LVR2506	2.2.1	ethionamide	4327363	Rv3854c	ethA	c.110-111insA	NC	no	
LVR2506	2.2.1	ethambutol	4243190	Rv3794	embA	c.-43G>C	3) Uncertain significance	yes	yes
LVR2827	4.3.3	fluoroquinolones	7570	Rv0006	gyrA	p.Ala90Val	1) Assoc w R (LEV/OFX, MXF)	yes (OFX), no (MFX)	yes (OFX), no (MFX)
LVR2827	4.3.3	rifampicin	761155	Rv0667	rpoB	p.Ser450Leu	1) Assoc w R	yes	yes
LVR2827	4.3.3	streptomycin	1472359	rrs	rrs	r.514a>c	1) Assoc w R	yes	yes
LVR2827	4.3.3	aminoglycosides	1473246	rrs	rrs	r.1401a>g	1) Assoc w R (AMK, KAN, CAP)	yes (AMK, KAN, CAP)	yes (AMK, KAN, CAP)
LVR2827	4.3.3	ethionamide,isoniazid	1673425	Rv1483	fabG1	c.-15C>T	1) Asoc w R (INH, ETO)	yes (INH), no (ETO)	yes (INH), no (ETO)
LVR2827	4.3.3	isoniazid	2155168	Rv1908c	katG	p.Ser315Thr	1) Assoc w R	yes	yes
LVR2827	4.3.3	ethambutol	4247469	Rv3795	embB	p.Tyr319Ser	1) Assoc w R	yes	yes
LVR2827	4.3.3	ethionamide	4326461	Rv3854c	ethA	p.Ile338Ser	3) Uncertain significance	no	no
LVR2827	4.3.3	pyrazinamide	2289058	Rv2043c	pncA	p.Pro62Thr	2) Assoc w R - Interim	yes	yes
LVR5434	2.2.1	rifampicin	761155	Rv0667	rpoB	p.Ser450Leu	1) Assoc w R	yes	yes
LVR5434	2.2.1	streptomycin	781687	Rv0682	rpsL	p.Lys43Arg	1) Assoc w R	NA	yes
LVR5434	2.2.1	isoniazid	2155168	Rv1908c	katG	p.Ser315Thr	1) Assoc w R	yes	yes
LVR5434	2.2.1	pyrazinamide	2289105	Rv2043c	pncA	p.Ala46Glu	2) Assoc w R - Interim	yes	yes
LVR5434	2.2.1	ethambutol	4247429	Rv3795	embB	p.Met306Val	1) Assoc w R	yes	yes
LVR3274	4.3.3	rifampicin	761155	Rv0667	rpoB	p.Ser450Leu	1) Assoc w R	yes	yes
LVR3274	4.3.3	streptomycin	1472359	rrs	rrs	r.514a>c	1) Assoc w R	yes	yes
LVR3274	4.3.3	ethionamide,isoniazid	1673425	Rv1483	fabG1	c.-15C>T	1) Asoc w R (INH, ETO)	yes (INH), no (ETO)	yes (INH), no (ETO)
LVR3274	4.3.3	isoniazid	2155168	Rv1908c	katG	p.Ser315Thr	1) Assoc w R	yes	yes
LVR3274	4.3.3	pyrazinamide	2289054	Rv2043c	pncA	p.Asp63Gly	2) Assoc w R - Interim	yes	yes
LVR3274	4.3.3	ethambutol	4248003	Rv3795	embB	p.Gln497Arg	1) Assoc w R	yes	yes
LVR3274	4.3.3	capreomycin						yes	no
LVR1189	4.3.3	rifampicin	761110	Rv0667	rpoB	p.Asp435Val	1) Assoc w R	yes	yes
LVR1189	4.3.3	streptomycin	1472359	rrs	rrs	r.514a>c	1) Assoc w R	yes	yes
LVR1189	4.3.3	aminoglycosides	1473246	rrs	rrs	r.1401a>g	1) Assoc w R (AMK, KAN, CAP)	yes (AMK, KAN, CAP)	yes (AMK, KAN, CAP)
LVR1189	4.3.3	ethionamide,isoniazid	1673425	Rv1483	fabG1	c.-15C>T	1) Asoc w R (INH, ETO)	yes (INH), no (ETO)	yes (INH), no (ETO)
LVR1189	4.3.3	isoniazid	2155168	Rv1908c	katG	p.Ser315Thr	1) Assoc w R	yes	yes
LVR1189	4.3.3	ethambutol	4247431	Rv3795	embB	p.Met306Ile	1) Assoc w R	yes	yes
LVR1189	4.3.3	pyrazinamide	2289167	Rv2043c	pncA	c.69-74del	NC	yes	no
LVR1552	2.2.1	rifampicin	761110	Rv0667	rpoB	p.Asp435Val	1) Assoc w R	yes	yes
LVR1552	2.2.1	streptomycin	781687	Rv0682	rpsL	p.Lys43Arg	1) Assoc w R	NA	NA
LVR1552	2.2.1	ethionamide,isoniazid	1673425	Rv1483	fabG1	c.-15C>T	1) Asoc w R (INH, ETO)	yes (INH), yes (ETO)	yes (INH), yes (ETO)
LVR1552	2.2.1	isoniazid	2155168	Rv1908c	katG	p.Ser315Thr	1) Assoc w R	yes	yes
LVR1552	2.2.1	pyrazinamide	2288935	Rv2043c	pncA	p.Tyr103His	1) Assoc w R	yes	yes
LVR1552	2.2.1	kanamycin, amikacin	2715342	Rv2416c	eis	c.-10G>A	1) Assoc w R (KAN); 3) Uncertain significance (AMK)	yes (KAN), no (AMK)	yes (KAN), no (AMK)
LVR1552	2.2.1	ethambutol	4243190	Rv3794	embA	c.-43G>C	3) Uncertain significance	yes	yes
LVR0142	2.2.1	rifampicin	761155	Rv0667	rpoB	p.Ser450Leu	1) Assoc w R	yes	yes
LVR0142	2.2.1	streptomycin	781687	Rv0682	rpsL	p.Lys43Arg	1) Assoc w R	yes	yes
LVR0142	2.2.1	isoniazid	2155168	Rv1908c	katG	p.Ser315Thr	1) Assoc w R	yes	yes
LVR0142	2.2.1	pyrazinamide	2289252	Rv2043c	pncA	c.-11A>G	1) Assoc w R	yes	yes
LVR0142	2.2.1	kanamycin, amikacin	2715342	Rv2416c	eis	c.-10G>A	1) Assoc w R (KAN); 3) Uncertain significance (AMK)	NA (KAN), no (AMK)	NA (KAN), no (AMK)
LVR0142	2.2.1	ethambutol	4243221	Rv3794	embA	c.-12C>T	1) Assoc w R	yes	yes
LVR0142	2.2.1	ethambutol	4247399	Rv3795	embB	p.Asn296His	3) Uncertain significance	yes	yes
LVR0142	2.2.1	ethionamide	4327363	Rv3854c	ethA	c.110del	1) Assoc w R	NA	NA
LVR5462	2.2.1	rifampicin	761155	Rv0667	rpoB	p.Ser450Leu	1) Assoc w R	yes	yes
LVR5462	2.2.1	streptomycin	781687	Rv0682	rpsL	p.Lys43Arg	1) Assoc w R	yes	yes
LVR5462	2.2.1	isoniazid	2155168	Rv1908c	katG	p.Ser315Thr	1) Assoc w R	yes	yes
LVR5462	2.2.1	pyrazinamide	2289105	Rv2043c	pncA	p.Ala46Glu	2) Assoc w R - Interim	yes	yes
LVR5462	2.2.1	ethambutol	4247429	Rv3795	embB	p.Met306Val	1) Assoc w R	yes	yes
LVR0758	4.3.3	rifampicin	761155	Rv0667	rpoB	p.Ser450Leu	1) Assoc w R	yes	yes
LVR0758	4.3.3	streptomycin	1472359	rrs	rrs	r.514a>c	1) Assoc w R	yes	yes
LVR0758	4.3.3	ethionamide,isoniazid	1673425	Rv1483	fabG1	c.-15C>T	1) Asoc w R (INH, ETO)	yes (INH), no (ETO)	yes (INH), no (ETO)
LVR0758	4.3.3	isoniazid	2155168	Rv1908c	katG	p.Ser315Thr	1) Assoc w R	yes	yes
LVR0758	4.3.3	pyrazinamide	2289054	Rv2043c	pncA	p.Asp63Gly	2) Assoc w R - Interim	yes	yes
LVR0758	4.3.3	ethambutol	4248003	Rv3795	embB	p.Gln497Arg	1) Assoc w R	yes	yes
LVR0758	4.3.3	capreomycin						yes	no
LVR3549	2.2.1	rifampicin	761155	Rv0667	rpoB	p.Ser450Leu	1) Assoc w R	yes	yes
LVR3549	2.2.1	streptomycin	781687	Rv0682	rpsL	p.Lys43Arg	1) Assoc w R	yes	yes
LVR3549	2.2.1	isoniazid	2155168	Rv1908c	katG	p.Ser315Thr	1) Assoc w R	yes	yes
LVR3549	2.2.1	pyrazinamide	2288853	Rv2043c	pncA	c.380-388del	1) Assoc w R	yes	yes
LVR3549	2.2.1	ethambutol						yes	no
LVR6749	2.2.1	rifampicin	761155	Rv0667	rpoB	p.Ser450Leu	1) Assoc w R	yes	yes
LVR6749	2.2.1	streptomycin	781687	Rv0682	rpsL	p.Lys43Arg	1) Assoc w R	no	no
LVR6749	2.2.1	isoniazid	2155168	Rv1908c	katG	p.Ser315Thr	1) Assoc w R	yes	yes
LVR6749	2.2.1	pyrazinamide	2288853	Rv2043c	pncA	c.380-388del	1) Assoc w R	yes	yes
LVR6749	2.2.1	ethambutol	4243190	Rv3794	embA	c.-43G>C	3) Uncertain significance	no	no
LVR1491	4.3.3	fluoroquinolones	7570	Rv0006	gyrA	p.Ala90Val	1) Assoc w R (LEV/OFX, MXF)	yes (OFX), no (MFX)	yes (OFX), no (MFX)
LVR1491	4.3.3	rifampicin	761155	Rv0667	rpoB	p.Ser450Leu	1) Assoc w R	yes	yes
LVR1491	4.3.3	streptomycin	1472359	rrs	rrs	r.514a>c	1) Assoc w R	yes	yes
LVR1491	4.3.3	aminoglycosides	1473246	rrs	rrs	r.1401a>g	1) Assoc w R (AMK, KAN, CAP)	yes (AMK, KAN, CAP)	yes (AMK, KAN, CAP)
LVR1491	4.3.3	ethionamide,isoniazid	1673425	Rv1483	fabG1	c.-15C>T	1) Asoc w R (INH, ETO)	yes (INH), NA (ETO)	yes (INH), NA (ETO)
LVR1491	4.3.3	isoniazid	2155168	Rv1908c	katG	p.Ser315Thr	1) Assoc w R	yes	yes
LVR1491	4.3.3	pyrazinamide	2289206	Rv2043c	pncA	p.Asp12Glu	1) Assoc w R	yes	yes
LVR1491	4.3.3	ethambutol	4247429	Rv3795	embB	p.Met306Leu	1) Assoc w R	no	no
LVR7091	2.2.1	fluoroquinolones	7582	Rv0006	gyrA	p.Asp94Gly	1) Assoc w R (LEV/OFX, MXF)	yes (OFX), NA (MFX)	yes (OFX), NA (MFX)
LVR7091	2.2.1	rifampicin	761155	Rv0667	rpoB	p.Ser450Leu	1) Assoc w R	yes	yes
LVR7091	2.2.1	rifampicin	764363	Rv0668	rpoC	p.Gly332Arg	3) Uncertain significance	yes	yes
LVR7091	2.2.1	streptomycin	781687	Rv0682	rpsL	p.Lys43Arg	1) Assoc w R	yes	yes
LVR7091	2.2.1	aminoglycosides	1473246	rrs	rrs	r.1401a>g	1) Assoc w R (AMK, KAN, CAP)	yes (AMK, KAN, CAP)	yes (AMK, KAN, CAP)
LVR7091	2.2.1	isoniazid	2155168	Rv1908c	katG	p.Ser315Thr	1) Assoc w R	yes	yes
LVR7091	2.2.1	ethambutol	4247399	Rv3795	embB	p.Asn296His	3) Uncertain significance	yes	yes
LVR7091	2.2.1	ethambutol	4247431	Rv3795	embB	p.Met306Ile	1) Assoc w R	yes	yes
LVR7091	2.2.1	ethionamide	4327363	Rv3854c	ethA	c.110del	1) Assoc w R	no	no
LVR7091	2.2.1	pyrazinamide	2288682	Rv2043c	pncA	c.380-388del	1) Assoc w R	yes	yes
LVR8181	2.2.1	fluoroquinolones	7570	Rv0006	gyrA	p.Ala90Val	1) Assoc w R (LEV/OFX, MXF)	no (OFX), NA (MFX)	no (OFX), NA (MFX)
LVR8181	2.2.1	rifampicin	761110	Rv0667	rpoB	p.Asp435Val	1) Assoc w R	yes	yes
LVR8181	2.2.1	streptomycin	781687	Rv0682	rpsL	p.Lys43Arg	1) Assoc w R	yes	yes
LVR8181	2.2.1	ethionamide,isoniazid	1673425	Rv1483	fabG1	c.-15C>T	1) Asoc w R (INH, ETO)	yes (INH), NA (ETO)	yes (INH), NA (ETO)
LVR8181	2.2.1	isoniazid	2155168	Rv1908c	katG	p.Ser315Thr	1) Assoc w R	yes	yes
LVR8181	2.2.1	pyrazinamide	2288935	Rv2043c	pncA	p.Tyr103His	1) Assoc w R	yes	yes
LVR8181	2.2.1	kanamycin, amikacin	2715342	Rv2416c	eis	c.-10G>A	1) Assoc w R (KAN); 3) Uncertain significance (AMK)	NA (KAN), yes (AMK)	NA (KAN), yes (AMK)
LVR8181	2.2.1	ethionamide	4327363	Rv3854c	ethA	c.110-111insA	NC	NA	NA
LVR8181	2.2.1	ethambutol	4243190	Rv3794	embA	c.-43G>C	3) Uncertain significance	yes	yes
LVR8181	2.2.1	capreomycin						yes	no
LVR6217	2.2.1	rifampicin	761109	Rv0667	rpoB	p.Asp435Tyr	1) Assoc w R	yes	yes
LVR6217	2.2.1	rifampicin	761166	Rv0667	rpoB	p.Pro454Ser	3) Uncertain significance	yes	yes
LVR6217	2.2.1	streptomycin	781687	Rv0682	rpsL	p.Lys43Arg	1) Assoc w R	yes	yes
LVR6217	2.2.1	aminoglycosides	1473246	rrs	rrs	r.1401a>g	1) Assoc w R (AMK, KAN, CAP)	yes (AMK, CAP), NA (KAN)	yes (AMK, CAP), NA (KAN)
LVR6217	2.2.1	isoniazid	2155168	Rv1908c	katG	p.Ser315Thr	1) Assoc w R	yes	yes

LVR6217	2.2.1	pyrazinamide	2289016	Rv2043c	pncA	p.Thr76Pro	1) Assoc w R	yes	yes
LVR6217	2.2.1	ethambutol	4247730	Rv3795	embB	p.Gly406Ala	1) Assoc w R	yes	yes
LVR6217	2.2.1	ethionamide	4327441	Rv3854c	ethA	c.32del	2) Assoc w R - Interim	NA	NA
LVR1816	4.3.3	rifampicin	761110	Rv0667	rpoB	p.Asp435Val	1) Assoc w R	yes	yes
LVR1816	4.3.3	streptomycin	1472359	rrs	rrs	r.514a>c	1) Assoc w R	yes	yes
LVR1816	4.3.3	aminoglycosides	1473246	rrs	rrs	r.1401a>g	1) Assoc w R (AMK, KAN, CAP)	yes (AMK, CAP), NA (KAN)	yes (AMK, CAP), NA (KAN)
LVR1816	4.3.3	ethionamide,isoniazid	1673425	Rv1483	fabG1	c.-15C>T	1) Asoc w R (INH, ETO)	yes (INH), NA (ETO)	yes (INH), NA (ETO)
LVR1816	4.3.3	isoniazid	2155168	Rv1908c	katG	p.Ser315Thr	1) Assoc w R	yes	yes
LVR1816	4.3.3	ethambutol	4247431	Rv3795	embB	p.Met306Ile	1) Assoc w R	yes	yes
LVR1816	4.3.3	pyrazinamide	2289069	Rv2043c	pncA	p.Phe58Cys	3) Uncertain significance	yes	yes
LVR7111	2.2.1	rifampicin	761110	Rv0667	rpoB	p.Asp435Val	1) Assoc w R	yes	
LVR7111	2.2.1	streptomycin	781687	Rv0682	rpsL	p.Lys43Arg	1) Assoc w R	yes	yes
LVR7111	2.2.1	ethionamide,isoniazid	1673425	Rv1483	fabG1	c.-15C>T	1) Asoc w R (INH, ETO)	yes (INH), yes (ETO)	yes (INH), yes (ETO)
LVR7111	2.2.1	isoniazid	2155168	Rv1908c	katG	p.Ser315Thr	1) Assoc w R	yes	yes
LVR7111	2.2.1	pyrazinamide	2288935	Rv2043c	pncA	p.Tyr103His	1) Assoc w R	yes	yes
LVR7111	2.2.1	kanamycin, amikacin	2715342	Rv2416c	eis	c.-10G>A	1) Assoc w R (KAN); 3) Uncertain significance (AMK)	no (KAN, AMK)	no (KAN, AMK)
LVR7111	2.2.1	ethambutol	4243190	Rv3794	embA	c.-43G>C	3) Uncertain significance	yes	yes
LVR9980	2.2.1	fluoroquinolones	7581	Rv0006	gyrA	p.Asp94Tyr	1) Assoc w R (LEV/OFX, MXF)	yes (OFX), yes (MFX)	yes (OFX), yes (MFX)
LVR9980	2.2.1	rifampicin	761109	Rv0667	rpoB	p.Asp435Tyr	1) Assoc w R	yes	yes
LVR9980	2.2.1	rifampicin	761166	Rv0667	rpoB	p.Pro454Ser	3) Uncertain significance	yes	yes
LVR9980	2.2.1	streptomycin	781687	Rv0682	rpsL	p.Lys43Arg	1) Assoc w R	NA	NA
LVR9980	2.2.1	aminoglycosides	1473246	rrs	rrs	r.1401a>g	1) Assoc w R (AMK, KAN, CAP)	yes (AMK, KAN, CAP)	yes (AMK, KAN, CAP)
LVR9980	2.2.1	isoniazid	2155168	Rv1908c	katG	p.Ser315Thr	1) Assoc w R	yes	yes
LVR9980	2.2.1	pyrazinamide	2289016	Rv2043c	pncA	p.Thr76Pro	1) Assoc w R	yes	yes
LVR9980	2.2.1	ethambutol	4247730	Rv3795	embB	p.Gly406Ala	1) Assoc w R	yes	yes
LVR9980	2.2.1	ethionamide	4327441	Rv3854c	ethA	c.32del	2) Assoc w R - Interim	yes	yes
LVR2221	4.3.3	rifampicin	761155	Rv0667	rpoB	p.Ser450Leu	1) Assoc w R	yes	yes
LVR2221	4.3.3	streptomycin	1472359	rrs	rrs	r.514a>c	1) Assoc w R	NA	NA
LVR2221	4.3.3	aminoglycosides	1473246	rrs	rrs	r.1401a>g	1) Assoc w R (AMK, KAN, CAP)	yes (AMK, CAP), NA (KAN)	yes (AMK, CAP), NA (KAN)
LVR2221	4.3.3	ethionamide,isoniazid	1673425	Rv1483	fabG1	c.-15C>T	1) Asoc w R (INH, ETO)	yes (INH), NA (ETO)	yes (INH), NA (ETO)
LVR2221	4.3.3	isoniazid	2155168	Rv1908c	katG	p.Ser315Thr	1) Assoc w R	yes	yes
LVR2221	4.3.3	pyrazinamide	2288868	Rv2043c	pncA	p.Val125Gly	2) Assoc w R - Interim	yes	yes
LVR2221	4.3.3	ethambutol	4247469	Rv3795	embB	p.Tyr319Ser	1) Assoc w R	yes	yes
LVR2221	4.3.3	ethionamide	4326461	Rv3854c	ethA	p.Ile338Ser	3) Uncertain significance	NA	NA
LVR3470	4.3.3	rifampicin	761155	Rv0667	rpoB	p.Ser450Leu	1) Assoc w R	yes	yes
LVR3470	4.3.3	streptomycin	1472359	rrs	rrs	r.514a>c	1) Assoc w R	NA	NA
LVR3470	4.3.3	ethionamide,isoniazid	1673425	Rv1483	fabG1	c.-15C>T	1) Asoc w R (INH, ETO)	yes (INH), yes (ETO)	yes (INH), yes (ETO)
LVR3470	4.3.3	isoniazid	2155168	Rv1908c	katG	p.Ser315Thr	1) Assoc w R	yes	yes
LVR3470	4.3.3	pyrazinamide	2289054	Rv2043c	pncA	p.Asp63Gly	2) Assoc w R - Interim	yes	yes
LVR3470	4.3.3	ethambutol	4248003	Rv3795	embB	p.Gln497Arg	1) Assoc w R	yes	yes
LVR3470	4.3.3	capreomycin						yes	no
LVR9235	4.3.3	fluoroquinolones	7581	Rv0006	gyrA	p.Asp94Asn	1) Assoc w R (LEV/OFX, MXF)	yes (OFX), NA (MFX)	yes (OFX), NA (MFX)
LVR9235	4.3.3	rifampicin	761155	Rv0667	rpoB	p.Ser450Leu	1) Assoc w R	yes	yes
LVR9235	4.3.3	streptomycin	1472359	rrs	rrs	r.514a>c	1) Assoc w R	NA	NA
LVR9235	4.3.3	ethionamide,isoniazid	1673425	Rv1483	fabG1	c.-15C>T	1) Asoc w R (INH, ETO)	yes (INH), NA (ETO)	yes (INH), NA (ETO)
LVR9235	4.3.3	isoniazid	2155168	Rv1908c	katG	p.Ser315Thr	1) Assoc w R	yes	yes
LVR9235	4.3.3	pyrazinamide	2289054	Rv2043c	pncA	p.Asp63Gly	2) Assoc w R - Interim	yes	yes
LVR9235	4.3.3	ethambutol	4248003	Rv3795	embB	p.Gln497Arg	1) Assoc w R	yes	yes
LVR9235	4.3.3	capreomycin						yes	no
LVR8846	2.2.1	rifampicin	761110	Rv0667	rpoB	p.Asp435Val	1) Assoc w R	yes	yes
LVR8846	2.2.1	streptomycin	781687	Rv0682	rpsL	p.Lys43Arg	1) Assoc w R	NA	NA
LVR8846	2.2.1	ethionamide,isoniazid	1673425	Rv1483	fabG1	c.-15C>T	1) Asoc w R (INH, ETO)	yes (INH), NA (ETO)	yes (INH), NA (ETO)
LVR8846	2.2.1	isoniazid	2155168	Rv1908c	katG	p.Ser315Thr	1) Assoc w R	yes	yes
LVR8846	2.2.1	pyrazinamide	2288935	Rv2043c	pncA	p.Tyr103His	1) Assoc w R	yes	yes
LVR8846	2.2.1	kanamycin, amikacin	2715342	Rv2416c	eis	c.-10G>A	1) Assoc w R (KAN); 3) Uncertain significance (AMK)	NA (KAN), no (AMK)	NA (KAN), no (AMK)
LVR8846	2.2.1	ethambutol	4243190	Rv3794	embA	c.-43G>C	3) Uncertain significance	yes	yes
